# Rhizosphere interface microbiome reassembly by arbuscular mycorrhizal fungi weakens cadmium migration dynamics

**DOI:** 10.1002/imt2.133

**Published:** 2023-08-31

**Authors:** Hong‐Rui Wang, Xin‐Ran Du, Zhuo‐Yun Zhang, Fu‐Juan Feng, Jia‐Ming Zhang

**Affiliations:** ^1^ College of Life Science Northeast Forestry University Harbin China

**Keywords:** arbuscular mycorrhizal fungi, cadmium, microbiome reassembly, rhizosphere, soil microbial interaction

## Abstract

The prevalence of cadmium (Cd)‐polluted agricultural soils is increasing globally, and arbuscular mycorrhizal fungi (AMF) can reduce the absorption of heavy metals by plants and improve mineral nutrition. However, the immobilization of the rhizosphere on cadmium is often overlooked. In this study, *Glomus mosseae* and *Medicago sativa* were established as symbiotes, and Cd migration and environmental properties in the rhizosphere were analyzed. AMF reduced Cd migration, and Cd^2+^ changed to an organic‐bound state. AMF symbiosis treatment and Cd exposure resulted in microbial community variation, exhibiting a distinct deterministic process (|βNTI| > 2), which ultimately resulted in a core microbiome function of heavy metal resistance and nutrient cycling. AMF increased available N and P, extracellular enzyme activity (LaC, LiP, and CAT), organic matter content (TOC, EOC, and GRSP), and Eh of the rhizosphere soil, significantly correlating with decreased Cd migration (*p* < 0.05). Furthermore, AMF significantly affected root metabolism by upregulating 739 metabolites, with flavonoids being the main factor causing microbiome variation. The structural equation model and variance partial analysis revealed that the superposition of the root metabolites, microbial, and soil exhibited the maximum explanation rate for Cd migration reduction (42.4%), and the microbial model had the highest single explanation rate (15.5%). Thus, the AMF in the rhizosphere microenvironment can regulate metabolite–soil–microbial interactions, reducing Cd migration. In summary, the study provides a new scientific explanation for how AMF improves plant Cd tolerance and offers a sustainable solution that could benefit both the environment and human health.

## Highlights

- Arbuscular mycorrhizal fungi (AMF) resulted in microbial community assembling to migration capacity.
- AMF promotes recruitment of metal‐resistant microbial community.
- AMF changes root metabolites to affect rhizosphere bacterial community assembly.
- AMF passivates rhizosphere Cd based on regulating ecological characteristics.

## Graphical abstract

Arbuscular mycorrhizal fungi (AMF) reduced Cd migration in the rhizosphere through microbial community variation, exhibiting a distinct deterministic process, which ultimately resulted in a core microbiome function of heavy metal resistance and nutrient cycling. The above process is related to upregulating root metabolites by AMF, with flavonoids, coumarin, fatty acids and so on being the main factor causing microbiome variation. Additionally, the superposition of the root metabolites, microbial, and soil make contributed to Cd migration reduction, and the microbial model had the highest single explanation rate. Thus, the AMF in the rhizosphere microenvironment can regulate metabolite‐soil‐microbial interactions, reducing Cd migration.

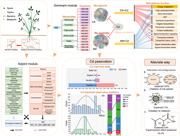

## INTRODUCTION

Cadmium (Cd) is easily absorbed by crops and threatens human health as it remains in the soil and cannot be degraded owing to its long half‐life [1]. According to the Agency for Toxic Substances and Disease Registry, the global average level of Cd pollution in agricultural soil is 0.01–2 mg/kg; however, the pollution rate in some areas exceeds this level. Therefore, Cd is the first harmful chemical substance of global significance [2]. Owing to the limited amount of cultivated land globally, the absorption of pollutants should be reduced while simultaneously improving crop resistance to stress [3]. The artificial inoculation of functional microorganisms effectively solves these problems [4]. Arbuscular mycorrhizal fungi (AMF) are rhizosphere microorganisms that exist widely in nature and can form symbiotes with more than 90% of plants. Importantly, AMF can maintain symbiotic relationships with plants in severely polluted soils and regulate the rhizosphere environment [5], improving plant mineral nutrition and reducing plant absorption and heavy metal (HM) accumulation [6]. The resistance of *Medicago sativa* for Cd considerably improves after inoculation with AMF and reduces Cd transfer from the soil to aboveground organs, which is of great significance for herbage harvested from aboveground parts [3]. AMF can cause the “Bio‐dilution” of HMs in vivo by increasing plant biomass to improve plant tolerance, but the concentration of HMs absorbed tends to increase owing to the improvement of root morphology and function [7]. The alfalfa root biomass was increased after AMF inoculation (compared with without inoculation). Importantly, the root surface area (potential absorption area) increased by 24% (*p* < 0.05) without any significant increase in total Cd content uptake (*p* < 0.05) [3]. Therefore, we speculate that the AMF regulation of rhizosphere microecological characteristics prevents the absorption of Cd by roots, which is the “first line of defense” against Cd absorption.

Cd absorbed by plants mainly occurs in the rhizosphere, which is considered one of the most complex ecological regions and hotspots in the fields of biology and ecology [8]. The rhizosphere microenvironment is related to the chemical characteristics of pollutants, as well as the occurrence region of plant–soil feedback [9], among which the living microbial community is a key hub for achieving the above interaction [10]. Rhizosphere microbiome is the main driver of soil material cycling and conversion and an important factor affecting Cd bioavailability [11]. Changes in the metabolic function of microbes can decrease Cd bioavailability by modulating soil properties (aggregates, water content, organic matter, Eh, and pH) and possible microbial core functions toward HM resistance [12]. This process is closely related to the species and function of the keystone (core microbial or dominant species) [13]. Previous studies have reported microbial community rebuilding by AMF in the plant rhizosphere [5, 14, 15]; however, its regulatory pathways and hub signals have not been clarified, although the stimulator is considered to be phytogenic rhizosphere secretion [16]. Root exudates maintain the interaction between plants and microorganisms as signals [17], and AMF inoculation can cause changes (composition and content) in host plant root exudates [18], such as the acceleration of the synthesis of plant organic oxides, vitamins, polyphenols, plant hormones, flavonoids, and other metabolites, eventually leading to variations in the microbial community [19]. Determining the hub components of root exudates that affect rhizosphere microbial construction is difficult [16]. Although defective mutants of key genes for specific secretion synthesis can be constructed, the secretion components affecting rhizosphere microbial construction are indistinguishable, and most plants cannot obtain the corresponding mutants [20]. Therefore, the relationship of metabolites associated with the microbial community assembly is difficult to identify. The joint application of the topological overlap matrix (TOM) algorithm of weighted correlation network analysis (WGCNA) and the important prediction of variables in the random forest model, identifying the “key” metabolites induce rhizosphere microbiome assembly and reflect the microbial community characteristics (keystone, dominant species, and Bray–Curtis distance) in the “rebuild” state [21]. In addition, root exudates directly impact the soil, further influencing the interactions with microorganisms. Thus, understanding the pathways involved in this complex rhizosphere system can contribute to revealing the mechanisms through which Cd is passivated.

High microbial biomass and activity in the rhizosphere make the root interface an important hot spot for carbon and nutrient cycling [22]. However, in previous standardized experimental procedures, soil microbial diversity or enzyme activity was measured after soil homogenization, disregarding the difference in microbial activity between hot and nonhot spot areas and also causing disturbance to the in situ rhizosphere microbial environment, resulting in a large experimental deviation. The root box method can obtain rhizosphere soil in situ, achieving undisturbed sampling and displaying migration characteristics [23]. This study established *Glomus mosseae* and alfalfa as a symbiotic system and the root box‐Cd^2+^ membrane blotting combined assay. The Cd properties in the rhizosphere environment were analyzed in situ, and changes in root metabolomics, soil physicochemical properties, and microbial characteristics in situ under Cd stress induced by AMF inoculation were compared. This study aimed to address the following questions: (1) whether AMF reduces the Cd migration by regulating the rhizosphere environment; (2) the role of rhizosphere metabolites in the phylogeny of microbial, and the “signaling substances (metabolites)” that trigger microbial community rebuilding; and (3) regulation of root metabolite–soil–microbial interactions by AMF in the rhizosphere to reduce Cd uptake by plants. Therefore, in the present study, we elucidated causal interactions between the rhizosphere microbiota, host metabolism variation, and soil factor influencing root absorbing Cd under Cd pollution.

## RESULTS

### AMF drives the reduction of migration of Cd in the rhizosphere

**Figure 1:**
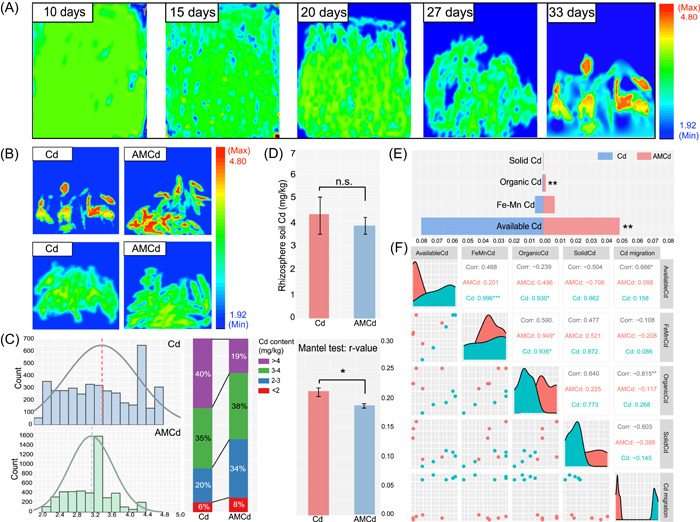
Migration and chemical states of Cd in the rhizosphere treated by Cd exposure and AMCd treatment. (A) Changes in Cd fluorescence imprinting in root boxes of groups treated for 0–33 days in Cd exposure. (B) After 33 days of Cd exposure, Cd fluorescence imprinting was performed on the rhizosphere of the Cd exposure and AMCd treatment group. (C) Frequency distribution of scanning values of fluorescent pixel sites in Cd and AMCd processing groups and colocation analysis based on Mantel test. (D) Cd content in rhizosphere soil of Cd exposure and AMCd treatment. (E) Chemical states of Cd in rhizosphere soil by Cd exposure and AMCd treatment. (F) Spearman correlation analysis between Cd chemical states and Cd migration characteristics (percentage of hot spots). Five biological replicates were performed for each index, and the error bars are standard errors. *Significant differences (*p* < 0.05) among each treatment. **Significant differences (*p* < 0.01) among each treatment. ***Significant differences (*p* < 0.001) among each treatment.

Soil Cd distribution showed a uniform distribution, gradual subsidence, and reduced distribution during 0–20 days of alfalfa transplantation into the root box (Figure 1A). Until Day 27, Cd was distributed along the plant roots, and hot spots of Cd accumulation appeared around the roots on Day 33 (red area, unit pixel Cd >4.05 mg/kg). However, the hotspot percentage in the AMCd group was significantly lower (*p* < 0.05) than that in the Cd‐exposed group (Figure 1B). Cd^2+^ fluorescence localization scanning also showed that the median frequency distribution of pixel sites and hotspot proportion of the AMCd group (larger than 4 mg/kg) were lower than those of the Cd exposure group (Figure 1C) (*p* < 0.05). Moreover, colocalisation analysis (image of root and Cd^2+^ fluorescence distribution) based on the Mantel test also revealed that Cd^2+^ aggregation in the rhizosphere after Cd exposure (*r* = 0.21, *p* = 0.032) was significantly higher than that after AMCd treatment (*r* = 0.17, *p* = 0.041) (*p* < 0.05). The Cd concentration in the Cd exposure (4.23 mg/kg) was higher than that in the AMCd treatment (3.92 mg/kg) in the rhizosphere (Figure 1D). In addition, the content of available Cd in the AMCd treatment was significantly lower by approximately 1/3 than that in Cd exposure (*p* < 0.05). However, the organic‐bound Cd increased significantly (*p* < 0.05), and the change of the organic‐bound Cd and available Cd had an evident correlation with Cd migration ability (Figure 1E,F).

### Changes in rhizosphere soil bacteria community

**Figure 2:**
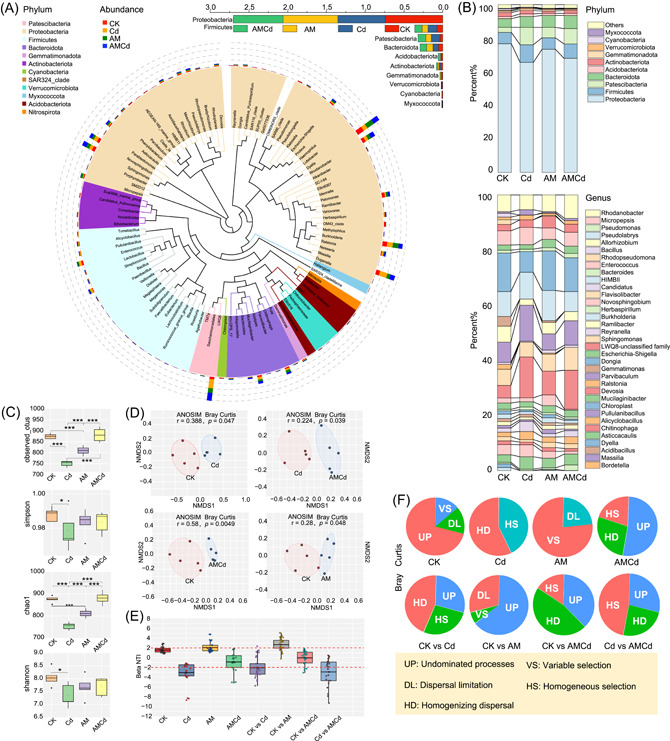
Composition and diversity (α and β) of alfalfa rhizosphere soil bacterial community under CK, Cd, AM, and AMCd treatments. (A) Maximum‐likelihood phylogenetic tree of the bacterial genome bins detected and the relative abundance of dominant phyla (top 10) in all soil samples under four treatments. (B) Bacteria in the soil at the phylum and genus levels under four treatments. (C) Indices of Shannon, Simpson, Chao1, and OTUs of bacteria in the rhizosphere soil. (D) Nonmetric multidimensional scaling (NMDS) ordinations based on Bray–Curtis distances and Bonferroni based on ANOSIM in rhizosphere soil bacterial communities. (E) Contributions of deterministic and stochastic processes in community assembly within collected rhizosphere soil samples. βNTI calculation of phylogenetic turnover among each treatment. (F) The relative influence of each community assembly process among four treatments was defined by the percentage of site pairs governed by each process. Five biological replicates were performed for each index, and the error bars are standard errors. *Significant differences (*p* < 0.05) among each treatment. **Significant differences (*p* < 0.01) among each treatment. ***Significant differences (*p* < 0.001) among each treatment.

First, 5187 OTUs were obtained by 16S sequencing (Supporting Information: Figure S2). Classification analysis showed that the rhizosphere bacteria mainly comprised six phyla (Figure 2A), of which Proteobacteria was the largest (67.65%), followed by Firmicutes (9.08%), Patescibacteria (8.06%), Bacteroidetes (7.36%), Actinobacteriota (2.08%), and Acidobacteriota (1.5%). Proteobacteria (61.67%) and Actinobacteriota (2.2%) decreased significantly after Cd exposure compared to CK (*p* < 0.05), whereas Firmicutes (11.72%), Patescibacteria (11.54%), Bacteroidetes (7.16%), Actinobacteriota (1.76%) were upregulated (*p* < 0.05). Proteobacteria (64.41%), Firmicutes (9.51%), and Bacteroidetes (8.67%) were more abundant in the AMCd‐treated group than in the Cd‐exposed group (*p* < 0.05). The top 45 genera which have the most relative abundance are shown in Figure 2B, the dominant being *Dyella* (30.59%), *LWQ8* (27.69%), *Burkholderia* (23.83%), *Massilia* (23.08%), *Rhodanobacter* (16.65%), *Sphingomonas* (16.44%), *Novosphingobium* (13.39%), *Enterococcus* (7.5%), *Bacillus* (5.7%), and *Micropepsis* (4.8%). Compared with CK, the abundance of *Dyella* and *Micropepsis* in the Cd exposure group decreased by 35% and 6.5%, respectively (*p* < 0.05). *Burkholderia*, *Massilia*, *Rhodanobacter*, and *Novosphingobium* increased (*p* < 0.05), and *Massilia* increased nearly threefold. In addition, *Dyella* and *Sphingomonas* in the AMCd treatment were significantly increased by 45% and 39%, respectively, compared to Cd (*p* < 0.05); however, Sphingomonas was higher than in CK (*p* < 0.05), and *Dyella* was not significantly different from CK. The rarefaction curve shows (Figure S3) that the Shannon, OTUs, Simpson, and Chao1 indices of the tested samples tended to be flat with increased sequencing quantity, indicating the high reliability of the results. In Cd exposure, Shannon, OTUs, Simpson, and Chao1 indices decreased significantly compared to those in CK (*p* < 0.05). Chao1 and OTUs in the AMCd treatment significantly improved with Cd exposure (*p* < 0.05), and the Shannon and Simpson indices increased slightly but not significantly (*p* > 0.05) (Figure 2C). Based on the paired comparison of Bray–Curtis distance and the complete data set of ANOSIM analysis (Figure 2D), CK versus Cd (*r* = 0.388, *p* = 0.047), Cd versus AMCd (*r* = 0.224, *p* = 0.039), CK versus AM (*r* = 0.280, *p* = 0.048), and CK versus AMCd (*r* = 0.580, *p* = 0.005). These results indicate that AM treatment and Cd exposure resulted in variations in the bacterial community. Microbial community assembly process analysis (Figure 2E) revealed that CK and AMCd treatments were random processes (|βNTI| < 2), and Cd and AM treatments were deterministic processes (|βNTI| > 2). Further, exploring the community change process (paired comparison βNTI) found that CK versus Cd, CK versus AM were |βNTI| > 2, and CK versus AMCd were |βNTI| < 2, Cd and AM were both the primary factors in the bacteria community assembly tending to the deterministic process; however, the former was for homogenous selection, the latter for variable selection (Figure 2F).

### Bacterial biomarker and keystone

**Figure 3:**
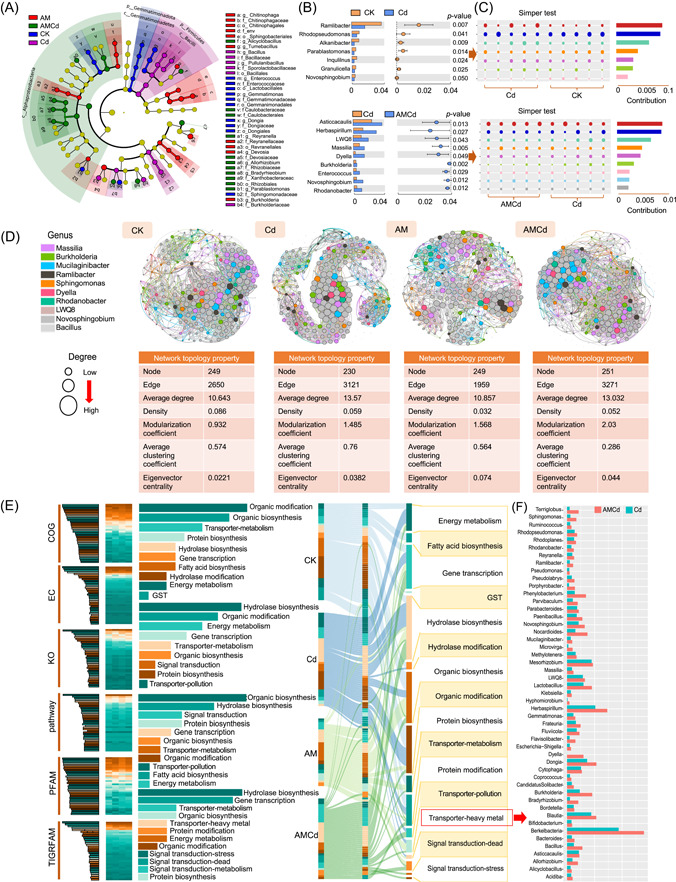
Prediction of keystone and function of alfalfa rhizosphere soil bacterial community under CK, Cd, AM, and AMCd treatments. (A) Bacteria biomarkers in the different treatments based on the LEfSe analysis. Different colors represent different treatments, and the circles from inside to outside correspond with phylum and genus. The color‐coded one within the cladogram denotes the taxa with a significantly higher relative abundance in the treatment as analyzed by the Kruskal–Wallis test with *p* < 0.05 and a logarithmic LDA score >3.5. Genera with a relative abundance of less than 0.1% were not included. (B) The main bacteria (genus level) differs significantly between CK versus Cd and Cd versus AMCd groups based on Welch's *t*‐test. (C) A similarity percentage (SIMPER) analysis based on the decomposition of the Bray–Curtis difference index between CK versus Cd and Cd versus AMCd groups. (D) Visual network and topology statistics of microbial co‐occurrence in the four treatments. (E) Cluster analysis of relative abundance based on PICRUSt2 functional annotation to soil bacteria under the four treatments. (F) Heavy metal (pollutant resistance) related 49 bacteria were selected in Cd versus AMCd treatment groups based on PICRUSt2 functional annotation, and their relative abundance was ranked.

The soil bacterial microbiome in the rhizosphere varied among the four treatments, as shown by LDA effect size (LEFSe) analysis (Figure 3A). The paired t‐test comparison showed that the significant differences in CK versus Cd were *Ramlibacter* and *Rhodopseudomonas* (Figure 3B) and that in Cd versus AMCd were *Asticcacaulis* and *Bacteroides*. Paired similarity percentage (SIMPER) analysis showed that the top five contributors of CK versus Cd and Cd versus AMCd were *LWQ8*, *Massilia*, *Dyella*, *Burkholderia*, and *Enterococcus* (Figure 3C). The topological features of the co‐occurrence network are shown in Figure 3D. The average network degrees of the CK and AM were 10.643 and 10.857, respectively, and the average weight degrees were 0.421 and 0.405, respectively. The average degree of Cd and AMCd were 13.57 and 13.032. The eigenvector centrality of the bacteria in the Cd exposure group was higher than CK, and the modularity coefficients were also increased. Compared with Cd, AMCd showed a higher modularity coefficient and eigenvector centrality, indicating a closer relationship between AMCd treatments. The highest scores of Page ranks or eigencentrality were identified as keystones, and *Proteobacteria*, *Bacteroidetes*, and *Firmicutes* with the highest ranking were identified as keystones (Supporting Information: Table S1). The keystone of CK was *Asticcacaulis*, *Methylobacterium*, and *Sphingomonas*, including nine genera (Page ranks = 0.007, Eigencentrality = 1), and that of Cd exposure was *Rhodanobacter*, *Dyella*, and *Burkholderia* in Cd exposure (Page ranks = 0.0058, Eigencentrality = 1) (Supporting Information: Table S1). *Parvibaculum*, *Massilia*, and *Alistipes*, among others, including eight keystone genera, were treated with AMCd (Page ranks = 0.0067, Eigencentrality = 1), and some keystones overlapped with CK (*LWQ8*, *Ramlibacter*) and Cd (*Massilia*). Thus, AMF and Cd can affect bacterial communities by recruiting preferred keystones.

### AMF recruits bacteria capable of passivating HM

Functional prediction of rhizosphere bacteria under the four treatments based on databases revealed that the four most abundant groups (accounting for 75%) were lipid metabolism, signal transduction, synthesis, and organic matter modification, and substance transport (Figure 3E). The functional preferences of rhizosphere bacteria were (from high to low) organic synthesis and modification, metabolite transport, hydrolytic enzyme synthesis, gene transcription, energy metabolism, and N absorption and assimilation in the CK. Hydrolases, organic synthesis and modification, energy metabolism, gene transcription, signal transduction, protein modification, and pollutant transport were observed upon Cd exposure. Meanwhile, the functions of stress signal transduction, programmed apoptosis signals, HM transport, and metabolite transport were increased in the AMCd treatment compared to Cd exposure. The microbial increase in the AMCd treatment with the HM transport (pollutant removal) function was selected. Approximately 139 bacteria could be negatively correlated with changes in Cd hotspots (*p* < 0.05), belonging to 101 genera (Supporting Information: Table S2), including *Dyella*, *LWQ8*, *Ruminococcus*, *Reyranella*, *Allorhizobium*, *Pseudolabrys*, *Novosphingobium*, and *Flavisolibacter*. Forty‐nine genera showed significant increases in abundance (Cd vs. AMCd; Figure 3F) and were compared using (Web of Science, CNKI, ELSEVIER, and KEGG). The above bacteria could phagocytose, adsorb, or embed HM; therefore, they were named HM‐removers.

### Soil factors associated with reduced Cd hot spots

**Figure 4:**
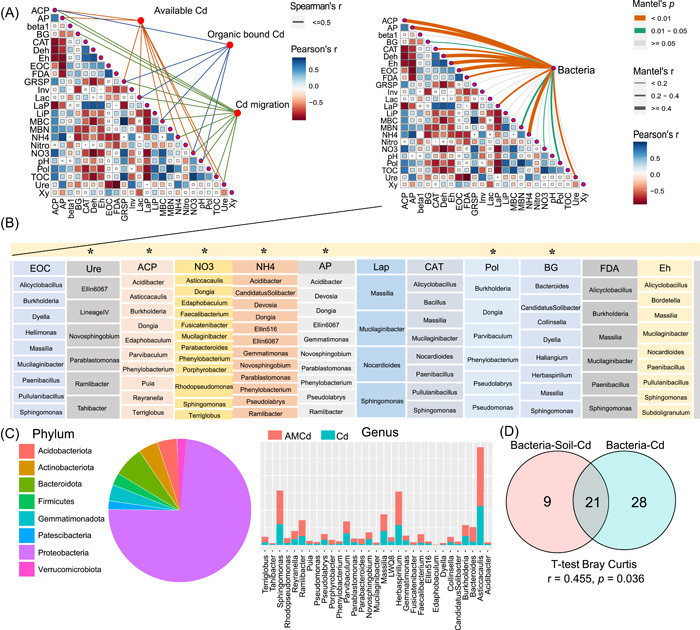
The interaction between soil and bacteria in the rhizosphere was investigated with “Cd–soil–bacteria” as the main axis. (A) Two Mantel tests of Cd property–soil property and bacteria–soil property, respectively (*p* < 0.05). (B) A Spearman correlation model between bacteria and soil factors, which both have Cd passivation ability. *Significant correlation (*p* < 0.05). (C) The selected bacteria were counted at phylum and genus levels, and their relative abundance under AM‐ and non‐AM treatment groups were compared. (D) A community similarity test based on Bray–Curtis distance for bacteria groups that can influence Cd properties and simultaneously affect Cd and soil.

Cd exposure reduced the available nutrient (N, P, and K) content in the rhizosphere soil compared with CK (*p* < 0.05) (Supporting Information: Figure S4); NH_4_‐N decreased by approximately 40% (*p* < 0.05), but EOC and GRSP were significantly increased (*p* < 0.05). However, AMCd treatment increased available N content (including NH_4_‐N and NO_3_‐N), available K, TOC, EOC, GRSP, and MBC compared to Cd exposure (*p* < 0.05). AMF improved rhizospheric soil nutrient concentration. In addition, the extracellular enzyme activity in the rhizosphere soil changed significantly. Compared with CK, Cd exposure significantly reduced enzyme activities related to N and P cycles in the rhizosphere soil (including acid phosphatase, laccase, urease, phenol oxidase, and nitrogenase, *p* < 0.05), whereas the C cycle‐related enzymes (β‐glucanase, β‐glucosidase, xylanase, leucine aminopeptidase, luciferase diacetate, and lignin peroxidase) were significantly increased (*p* < 0.05) (Supporting Information: Figure S5). Compared with Cd, the activities of N and P cycle‐related enzymes (including acid phosphatase, laccase, urease, phenol oxidase, and nitrogenase) in the AMCd treatment were increased, *p* < 0.05), whereas β‐glucanase, luciferase diacetate, and lignin peroxidase in the above C cycle related enzymes were decreased (*p* < 0.05). However, they all were higher than CK (*p* > 0.05). These results indicate that AMF improved the rhizospheric soil nutrient concentration by increasing extracellular enzyme activity (Supporting Information: Figure S6). Mantel test and the Spearman correlation model were used to screen the soil factors and bacterial groups related to Cd passivation based on “Cd‐soil attributes‐microorganisms” as the main axis (threshold: *r* > 0.6, *p* < 0.05). Acid phosphatase, urease, NO_3_‐N, NH_4_‐N, available P, β‐glucanase, and phenol oxidase may be related to the Cd passivation process (Figure 4A), and the above factors were significantly correlated with bacteria (Figure 4B). Acidobacteriota, Actinobacteriota, Bacteroidota, Firmicutes, Gemmatimonadota, Patescibacteria, Proteobacteria, and Verrucomicrobiota were significantly correlated with the above six soil factors (Figure 4C), and AM inoculation significantly increased the abundance of the above bacteria (Cd vs. AMCd). Approximately 63% of these bacteria were HM‐removers and influence soil and Cd migration indirectly or directly (Figure 4D). Therefore, changes in soil properties can reduce Cd migration.

### Changes in root metabolites and their effects on rhizosphere microecology

**Figure 5:**
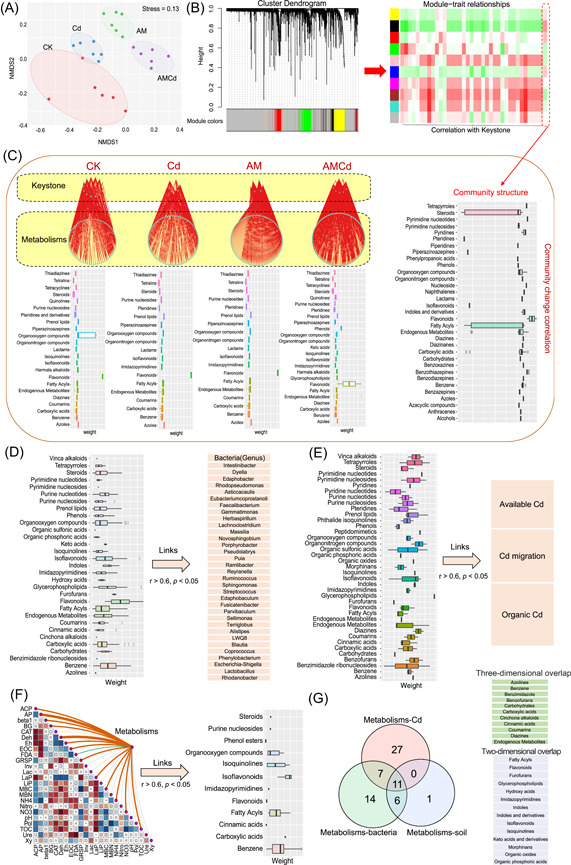
Root metabolome and rhizosphere microbiome interactions. (A) Nonmetric multidimensional scaling (NMDS) ordinations based on Bray–Curtis distances and Bonferroni based on ANOSIM in alfalfa root metabolisms. (B) The module division of root metabolites, the hierarchical clustering tree with module identification, the allocated modules, and the metabolite information contained in the modules. The colors red to green indicate the correlation between metabolite modules and microbial information from strong to moderate, among which only red represents a significant influence (*p* < 0.05). (C) A Spearman correlation model of the bacterial keystone and the Bray–Curtis distance information representing the community characteristic structure under four treatments and weight ranking of high effects metabolites were obtained by prediction. Spearman's correlation was calculated by relating the eigenmetabolite of each module to the relative abundance of each taxon using the WGCNA trait function. (D) A Spearman correlation model (*r* > 0.6, *p* < 0.05) between HM‐resistant bacteria and metabolite, which both increased by AM inoculation (AM vs. non‐AM, *t*‐test, *p* < 0.05) and a metabolite weight ranking. (E) A Spearman correlation model (*r* > 0.6, *p* < 0.05) between Cd property and metabolite, which increased by AM inoculation (AM vs. non‐AM, *t*‐test, *p* < 0.05) and a metabolite weight ranking. (F) A Mantel test (*r* > 0.6, *p* < 0.05) between soil property and metabolite, which increased by AM inoculation (AM vs. non‐AM, *t*‐test, *p* < 0.05) and a metabolite weight ranking. (G) A Venn diagram based on the statistics of the frequent occurrence of the same metabolites in the three groups of metabolic versus soil, metabolic versus Cd, and metabolic versus bacteria. WGCNA, weighted correlation network analysis.

Root metabolomics showed significant differences under different treatments (*p* < 0.05; Supporting Information: Figure S7A), with the Cd versus AMCd treatment group showing the largest difference (containing 301 different metabolites), followed by the AM versus AMCd treatment group (containing 235 different metabolites). Based on the ANOSIM analysis of the Bray–Curtis distance in the four treatments, AMF principally caused differences in metabolic substances (top Bray–Curtis ranks, Supporting Information: Figure S7B). In addition, it may upregulate the metabolic substance content through flavonoid synthesis, amino acid metabolism, phenyl propyl synthesis, and secondary metabolism (based on the comparison between the AM and non‐AMCd groups, Supporting Information: Figure S7C). Moreover, as mentioned above, AMF can affect the bacterial community to decrease Cd migration; therefore, weighted metabolite–microbial coexpression network analysis was used to explore the main factors affecting microbial community variation. The metabolites were divided into modules by hierarchical clustering (module corresponding color), and the model was adapted to microbial keystone and overall microbial community expression (model parameters: correlation >0.2, *p* < 0.05) (Figure 5A,B). The keystone and community matrix (Bray–Curtis distance) of microorganisms under different treatments were closely correlated with flavonoid metabolites (weight and module connectivity were highest) (Figure 5C). Therefore, flavonoid metabolites were considered the key hubs affecting community change, and AMF changed the bacterial community by increasing the flavonoid content (Figure 5C). In addition, using weighted metabolite–microbial coexpression network analysis, Spearman correlation model, and Mantel test, metabolites that can affect bacterial and soil properties (screened for Cd passivation ability) and reduce Cd migration were identified (Figure 5D–F). Eleven metabolites had ternary overlap, and 14 substances had binary overlap, indicating that metabolites, microbial, and soil properties could directly passivate Cd and had complex interactions among them (Figure 5G).

### AMF passivates rhizosphere Cd based on the regulation of metabolic–microbial–soil

**Figure 6:**
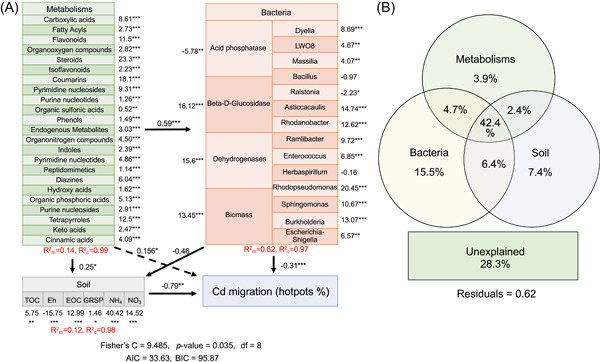
Process of cadmium passivation by AMF‐alfalfa symbiont. (A) Structural equation model of the interaction between multidimensional data sets of metabolites, microorganisms, soils, and Cd. (B) Variance decomposition analysis of the effect of metabolites, microbial, and soil on Cd migration. *Significant differences (*p* < 0.05) among each treatment. **Significant differences (*p* < 0.01) among each treatment. ***Significant differences (*p* < 0.001) among each treatment. AMF, arbuscular mycorrhizal fungi.

A composite SEM clarified the interactions among root exudates, soil factors, microbes, and Cd (Figure 6A). After AMF inoculation, Cd migration capacity was reduced in three ways, “metabolite–microbe–soil–Cd,” and “metabolite–soil–Cd,” two indirect actions, and “metabolite–Cd“ direct action mode. Complementary VPA found that the combined effect of the three could reduce the maximum explanation rate of Cd migration (42.4%), suggesting the importance of combining the three for Cd toxicity mitigation (Figure 6B). Regarding the single interpretation rate, the microbial population was the largest (15.5%), whereas the total explanation rate of microbes (including single microbes and their superimposed effects) reached 72%, verifying that rhizosphere microbial remodeling played an important role in reducing Cd migration. The rhizosphere soil is affected by root metabolites and soil microbial secretions; therefore, soil extracellular enzymes (LaC, LiP, and CAT), organic matter (TOC, EOC, and GRSP), and the REDOX potential are the key cadmium passivation factors (Figure 4A–C). Based on our results, we can fully understand the interactions of metabolites and soil microbes in the rhizosphere as well as minimize the risks of collinearity and pseudo‐correlation, which will help resolve how AMF regulates Cd in rhizosphere microspheres.

## DISCUSSION

### Recruitment of “functional community” by rhizosphere bacteria rebuilding

Using ANOSIM analysis, it was found that AM and Cd‐induced rebuilding of the rhizosphere microbial community (Figure 2D). Furthermore, the AMCd treatment group was formed by 49 genera of bacteria composed of HM‐remover (Figure 3E). This is one of the reasons for the Cd passivation of microorganisms. Therefore, how are these resistant microbial communities formed? We first analysed whether the AM experimental group (no Cd exposure) could also recruit function microbial. A total of 32 common rhizosphere growth‐promoting bacteria (AM vs. non‐AM) were recruited by AMF, of which 19 were nitrogen fixing, 10 were phosphorus solubilizing, and three had both capacities (Supporting Information: Figure S8). This explains why AM inoculation can effectively alleviate the N and P limitations caused by Cd pollution in rhizosphere soil (Supporting Information: Figure S4–S6). The Spearman correlation model (*r* > 0.6, *p* < 0.01), random forest model prediction (Importance > ShadowMax), and HM‐remover in the inoculation treatment group were obtained (Figure 3F), of which 10 were plant growth‐promoting rhizobacteria (PGPR) (Supporting Information: Table S3). Their mechanisms of action on HM are divided into three categories based on the following studies. The first category includes the phagocytosis of HM (Supporting Information: Table S4), which has 19 groups, including *Bacteroides*, *Burkholderia*, and *Candidatus solibacter* [24, 25, 26, 27, 28, 29, 30, 31, 32, 33, 34, 35, 36, 37, 38, 39, 40, 41], can directly ingest (phagocytose) HM ions. The second has surface adsorption ability (Supporting Information: Table S4), including 15 groups such as *Asticcacaulis*, *Bacillus*, and *Bacteroides*. Abundant functional groups, such as –OH, C═O, and –NH existed on the biofilm surface of the above bacteria, which could be directly complexed with Pb, Cu, Hg, Cd, and other bivalent HM ions [42, 43, 44, 45, 46, 47, 48, 49, 50, 51, 52, 53, 54, 55]. Therefore, this indicates that organic‐bound Cd increased (Figure 1E). The third achieves Cd degradation through secretion production, including 10 bacteria of *Acidibacillus*, *Alicyclobacillus*, and *Allorhizobium* (Supporting Information: Table S4). *Acidibacillus*, *Alicyclobacillus*, and *Bordetella* secretions reduce metal ions to mineral‐bound and residual states [56, 57, 58]. While *Parvibaculum*, *Phenylobacterium*, and *Porphyrobacter* produce extracellular polymers, metallothionein, and porphyrins, respectively, which are all strong metal complexes [28, 59, 60, 61, 62, 63, 64]. These microbes indirectly decrease Cd migration by altering soil properties (extracellular enzymes, GRSP, MBC, and Eh). Thus, the recruited “functional community” reduced Cd migration in several ways.

### Nonaccidental behavior by AMF recruitment of resistance microbial community

AMF can recruit PGPR [65]; however, the randomness of the recruitment of HM‐removing bacteria requires verification. Therefore, a meta‐analysis was conducted by integrating literature on the relationship between AMF and rhizosphere bacteria into 34 parallel experiments (The methods and results are detailed in the supplementary materials, and references are available at Supporting Information: Table S5). The screening of Cd‐resistant bacteria in the above literature and the HM‐remover strains in this study mostly coincided (Supporting Information: Table S6). Among them, 33 taxa, including *Rhodoplanes*, *Burkholderia*, and *Sphingomonas*, frequently appeared (ranking in the top 25%) (Supporting Information: Figure S9). Indigenous microbial species (OTUs) were the main cause of the frequency differences (*p* < 0.05) (Supporting Information: Figure S10), and the random forest model results were also consistent with the above (Supporting Information: Figure S11). Specifically, there was a significant positive correlation between the two (*r* = 0.53, *p* = 0.0011) (linear fitting model, Supporting Information: Figure S12), and the regulation was consistent with grassland, garden, farmland, marsh, and wasteland ecosystems (*p* < 0.05) (Supporting Information: Figure S13). This effectively proved that AMF could recruit HM‐resistant bacteria, but the number and type of recruitment depended on the species of indigenous microorganisms (OTUs), and this explains the difference in coincidence rates (*p* < 0.05) (Supporting Information: Figure S10).

### Conditions for metal‐resistant bacteria play a key function

Although the above analysis proved that resistant bacteria recruited by AMF were universal, this did not mean an evident HM‐resistant functional microbiome. In contrast, some studies suggested that AMF could promote bacteria involved in nitrification and denitrification processes, nitrogen fixation, and nodulation to become keystones. This is because most of the experiments were based on AMF inoculation when the soil was poor in N and P, ultimately making the core function of the rhizosphere microbiome incline to the nutrient acquisition strategy [14, 15, 66, 67, 68, 69, 70, 71, 72, 73, 74, 75, 76, 77, 78, 79]. Therefore, they play an important role only when environmental stress causes the recruited functional microbes to become keystones. After AMF randomly recruiting bacteria, most bacteria have functional redundancy, and when encountering various stresses, functional microbial relief plays a leading role, making them keystones [80]. Simultaneously, if the stress of adversity is too high, the recruitment process will be accompanied by the death of bacteria, and only resistant microbes with strong vitality will be retained [81]. Similar to our study, AMF and Cd could recruit their preferred keystones (Figure 3D). In the AMCd treatment, keystone bacteria were increased, such as *Parvibaculum*, *Alistipes*, *Lactobacillus*, *Duganella*, *Ramlibacter*, *Bacteroides*, and *LWQ8*, based on AM inoculation (compared to the AM treatment group) (Supporting Information: Table S1). These seven bacteria belong to HM‐remover (Supporting Information: Table S2), which may be one of the reasons for the HM‐remover feature group in AMCd. Meanwhile, through *ggClusterNet*, modules related to Cd migration and chemical state change were clearly distinguished, and the above keystone was also an important hub of modules (Supporting Information: Figure S14). In conclusion, the combination of AMF's extensive recruitment of microorganisms and Cd's screening effect on them formed a functional module of HM resistance in the AMCd treatment.

### Root metabolites affect rhizosphere bacterial community assembly

Plants can release 20%–40% of photo‐assimilated carbon through root secretions [17], and the composition and content are related to macromolecular metabolites and passively diffused low‐molecular‐weight compounds produced by plant metabolism [16]. Specific metabolites of plant roots (coumarins, flavones, and benzene) as secretions affect the rhizosphere microbiome and the specific species enrichment [20]. In this study, weighted metabolite–microbial coexpression network analysis was used for prediction, and sugar, flavone, coumarin, phenol, fatty acids, and amino acids were significantly correlated with the microbial community composition (Bray–Curtis distance) and keystones (Supporting Information: Figure S7A–C). Based on the random forest model, flavonoids had the greatest influence on the dominant microbial species and keystones (the largest weight). Moreover, supplementary experiments have confirmed that it can be secreted into the extraneous environment in large quantities (Supporting Information: Figure S15). Therefore, flavonoids are signaling regulatory factors that affect bacterial community reassembly. In particular, phytogenic flavonoids regulate rhizosphere microbial communities [82]. The interactions between plants and microorganisms occur in two stages. First, plants secrete chemical signals into the rhizosphere that can encourage, limit, or inhibit microbial activity and proliferation. Flavonoids are often accepted as signaling molecules [82] and can selectively promote the growth and propagation of PGPR (such as *Azotobacter*) [83]. The keystone and dominant species in the AMCd treatment were mostly PGPR (including HM‐remover), possibly owing to the high contribution of flavonoids (Figure 5C). The second stage is a quorum sensing (QS) process, in which microbes detect low‐molecular‐weight compounds released by plant roots (or other microorganisms) and regulate various microbial population activities through a signal cascade [20]. Phytogenic flavonoids may be the regulatory molecules in the AHL family signaling pathways (AHL‐mediated QS system is the first “language family”) [17]. Moreover, phylogenetic lipids, phenols, alcohols, ketones, aldehydes, keystones, and terpenes are used as QS signaling substances to comprehensively regulate microbial communities [84], consistent with our prediction of metabolite species (Figure 5C–F). Polypeptides, coumarins, fatty acids, benzene, phenols, secondary metabolites, and other substances contributed significantly to microbiome assembly (*p* < 0.01). The above can be effectively secreted into the extraneous environment (Supporting Information: Figure S15). In summary, AMF regulates rhizosphere microbial rebuilding through the superimposed effect of flavonoid metabolites as the dominant metabolites accompanied by other small molecules. Although the model was used to predict the relationship between metabolites and microorganisms (high threshold background), accurate single/combination addition of metabolites is required to verify the relationship between the two.

## CONCLUSION

The study indicates that controlled metabolic activities in alfalfa roots are critical for beneficial interactions with the rhizosphere microbiota. Under Cd toxicity, AMF specifically assembles bacteria of the taxon HM‐resistant microbiome in the rhizosphere, and this in turn decreases Cd migration. A full understanding of the causal relationships between the rhizosphere microbiota, host metabolism regulation, and soil factors change could offer a sustainable solution that could benefit both the environment and human health. Based on our results, the flavones increased by AMF in the alfalfa rhizosphere might recruit HM‐remover bacteria. Hence, it provides a new scientific explanation for how AMF improves plant Cd tolerance from the perspective of plant and microbial ecological interactions.

## METHODS

### Alfalfa growth conditions

The topsoil (0–20 cm) was collected from soil (Cd background content of 0.017 mg/kg) of a corn field (126°48′E, 45°16′N), passed through a 2 mm sieve, and mixed with sterilized flower soil and vermiculite at 3:3:1 to form a composite medium. Furthermore, 50 g/kg activated and inactivated AMF bacterium (*G. mosseae* L.) (provided by Laboratory of Restoration Ecology, Heilongjiang University) were added to form two treatments: a control (CK) and an AMF inoculation, approximately 35 spores/g soil. They were placed into self‐made root boxes, and young alfalfa (*M. sativa* L.) seedlings (courtesy of the Chinese Academy of Agricultural Sciences) were planted in each box. The root box comprised a (polypropylene) PP plate with 20 × 20 × 0.3 cm dimensions. The door was movable, and the bottom was made of 600‐mesh nylon mesh. The soil in the root box absorbed water from the bottom up such that the relative position of soil Cd could not sediment with the flow. The root box was tilted at 45–50° to enable the roots to fit the door at the back of the box owing to ground growth, as shown in Supporting Information: Figure S1A–C. The root infection rate was determined every 10 days during seedling growth. Infection was considered successful when the infection rate reached 90% (Supporting Information: Figure S1D,F). Then, the two groups were treated with 2 mg/kg CdCl_2_ (the concentration obtained according to supplement materials) and sterile distilled water, and a total of four groups were formed: CK, Cd, AM, AMCd, and cultured for 25 days.

### Rhizosphere Cd fluorescence imprinting

The experimental process of root box in situ imprinting is shown in Supporting Information: Figure S1E (applied for a patent in the State Intellectual Property Office: CN114518347A) [85]. Root boxes treated with Cd and AMCd were randomly selected every 5 days for the Cd^2+^ fluorescence imprinting test. A standard 600‐mesh nylon film was cut to an appropriate size and soaked in LeadmiumTM fluorescent dye (Molecular Probes^TM^; Thermo Fisher Scientific) for approximately 1 h. The root box was opened, and the saturated membrane was tightly fitted onto the surface, covered, and returned. Approximately 0.5 h later, the root box was opened, the imprinted membrane was removed, and sand and mud particles on the surface were carefully removed using tweezers. It was then placed on an ultraviolet imager (GelDoc XR Biorad; Bio‐Rad Laboratories), and the SYBR Green fluorescence imaging mode was selected for imaging and taking photos. Digital images were analyzed using the ImageJ software 1.53 (MathWorks). An RGB image was converted into black and white, and each pixel was assigned a grayscale value corresponding to 0 for black and 256 for white. The average gray value of each circular calibration film was calculated. The Cd concentration in the calibration film is a function of the gray values. This function was applied to digital images of the fluorescence spectrum. To illustrate the results explicitly, we used color to describe the value of the gray image, where red corresponded to the highest gray value (value 256), and blue corresponded to the lowest gray value (value 0). The color area 25% higher than the average gray value of the pixel points was set as red, indicating the hotspot of Cd aggregation [23]. The Cd speciations (exchangeable Cd, carbonate‐bound Cd, Fe‐Mn oxides Cd, organic matter‐bound Cd, and residual Cd) in rhizosphere soil were analysed using Tessier's sequential extraction procedure [86]. Cd content (including different speciations) was determined using a Perkin Elmer 900 T atomic absorption UV spectrophotometer (Perkin Elmer).

### Measurement of soil properties

Rhizosphere soil sampling is shown in Supporting Information: Figure S1G. Soil pH values were measured using a pH meter with a soil‐water ratio of 1:5 (PHS‐3C; LEICI). Organic matter content was determined by an element analyser (Vario EL‐III; Elementar), and total carbon and total nitrogen (N) contents were determined by a dry combustion carbon and nitrogen analyser (JC‐NY4B; Jingcheng Instrument). Soil nitrate (NO_3_^−^‐N) and ammonia (NH_4_^+^‐N) were analyzed using Lachat 8000 flow injection analyser (Lachat QC5000; LACHAT). The soil oxidation–reduction potential (REDOX) was determined using the platinum electrode‐reference electrode method. The above methods refer to Xu et al. [86]. The glomalin‐related soil protein (GRSP) content was determined using the Battini et al. [87] method. Acid phosphatase, β‐1,4‐glucanase, β‐glucosidase, xylanase, catalase, dehydrogenase, invertase, laccase, urease, polyphenol oxidase, leucine aminopeptidase, lignin peroxidase, and FDA hydrolase were determined by microplate method. The above methods refer to Wang et al. [88]. Soil chemical properties were recorded using Excel (v.2019), and significance was tested using SPSS (v.19) analysis of variance.

### Extraction and polymerase chain reaction amplification of genomic DNA from rhizosphere soil bacteria

Total microbial community DNA was extracted from the samples using a Fast DNA Spin Kit for Soil (MP, Biomedicals). The bacterial 16S rRNA gene fragments were amplified with the universal primers 799F (5′‐GTGCC AGCMGCCGCGGTAA‐3′) and 1193R (5′‐CCCCGYCAATTCMTTTRAGT‐3′) fused with unique barcode [89]. Gel‐purified polymerase chain reaction products were mixed with equal molar following Illumina sequencing using the platform of Novaseq. 6000. All sequence data have been submitted to the National Center for Biotechnology Information (NCBI) Sequence Read Archive under Bioproject PRJNA975279.

### Microbial informatics analysis

QIIME2 software 2017.6 was used to calculate amplicon sequence variant (ASV), Shannon, Simpson, and Chao1 indices, and a Rarefaction Curve was drawn. QIIME2 software was used to calculate the Unifrac distance and plot the nonmetric multidimensional scaling (NMDS) dimension reduction graphs using packages ade4 and ggplot2 in R software. Subsequently, the ANOSIM function in the QIIME2 software was used to analyse significant differences between the groups' community structures. Finally, species with significant differences between the groups were analyzed using LEfSe or R software. LEfSe analysis was performed using LEfSe software and LDA threshold value should be greater than 3.5. By default, the linear discriminant analysis score threshold was four. R software was used to test the differences in the MetaStat analysis between the two comparison groups at six classification levels: phylum, class, order, family, genus, and species, and *p*‐values were obtained. Species with a *p* < 0.05 were identified as significantly different species. The *t*‐test also used R software to analyze significant differences in species at various taxonomic levels. ASV for microbes was conducted using PICRUSt2. The gene function spectrum was inferred from the gene information of the tree and ASV, and the gene function spectra of other unknown species in the Greengene database were used to construct the gene function prediction spectrum for the entire lineage of the bacterial domain. Finally, the bacterial community composition obtained by sequencing was “mapped” to a database to predict bacterial metabolic functions [90]. The bacterial network topology parameters were calculated using Cytoscape v 3.8.0 and Gpehi v 0.9.2.

### Correlation analysis of microbial‐environmental factors

Spearman correlation analysis was conducted between species and environmental factors, their significance was evaluated using Corr. test function in the R *Psych* package, and visualization was performed using the *Pheatmap* function. The vegan package in R was used for the Mantel test. Based on the species matrix and the provided environmental factor data matrix, the *Vegdist* function was first used to convert the distance matrix of the two types of data, and then the Mantel function was used to conduct Spearman correlation analysis on the two types of matrices to obtain *r* and *p*‐values [21].

### Determination of root metabolites

An appropriate amount of sample was accurately weighed into a 2 mL centrifuge tube, 600 μL MeOH (stored at −20°C) was added containing 2‐amino‐3‐(2‐chloro‐phenyl)‐propionic acid (4 ppm) and vortexed for 30 s. Glass beads (100 mg) were added, and samples were placed in a tissue grinder for 90 s at 60 Hz. After centrifugation for 10 min at 12,000 rpm and 4°C, the supernatant was filtered by a 0.22 μm membrane and transferred into a detection bottle for LC‐MS detection. The LC analysis was performed using a UHPLC System (Vanquish; Thermo Fisher Scientific). Mass spectrometric detection of metabolites was performed on Q Exactive (Q Exactive; Thermo Fisher Scientific) with an ESI ion source [91].

### Host root metabolome correlates with changes in the microbiome

We used the R package based on WGCNA to replace gene expression in metabolite ion scanning lineages with data sources to identify correlations between quantitative data sets: (1) Metabolite coexpression modules between alfalfa roots under four treatments; (2) Quantitative value of modular characteristic metabolites–microbial community association and in‐module hub metabolites. WGCNA is an unsupervised assay that clusters metabolites based on the expression profiles of 180 substance‐specific samples. To construct coexpression networks robustly, we reserved chromatographic peak areas of ≥5 mapped reads in corresponding regions of at least one substance. The substance expression was normalized to log_2_ (FPKM + 1). Soft threshold power β calculated adjacency. To minimize the effects of noise and false associations, we converted adjacency into a TOM and calculated the corresponding dissimilarity (dissTOM) as 1‐TOM. We used dissTOM as a distance measure for hierarchical clustering and set the minimum module size (number of genes) to 28 to detect the modules. To quantify the coexpression similarity of all modules, the characteristic genes were calculated and clustered according to their correlations, which were subsequently used to study the crosstalk between metabolite expression differences and microbial traits. We chose 0.8 value relatives for the cluster module. Modular characteristic metabolites were used to represent the patterns of substance expression within a module. The relative abundance of refined bacterial operational taxonomic unit (OTU) tables with taxonomic information was further imported into the WGCNA data framework, and each taxonomic taxon was regarded as a “feature interval.” The module characteristic metabolites were most significantly correlated with these microbial traits. Then, a paired comparison of the expression values of all modular characteristic genes of Pearson's correlation and their associated *p*‐values was generated. Bonferroni adjustment was used to correct multiple comparisons. We further studied those modules highly correlated with microbial taxa to identify the types of highly connected module ‘hub’ metabolites associated with microbial taxa. The above method can be referred to Yu et al. [16]. Rhizosphere secretion extraction experiments were conducted to verify that the key compounds identified above can be released into the extracellular environment in large quantities. See supplementary material for the methods and results.

### Relationship between metabolites, microbes, soil factors, and the Cd hot spots

Spearman and Mantel tests were separately used to determine the correspondence between the percentage of metabolite‐Cd aggregation hotspots and metabolite‐soil factors, and the threshold was set as *r* > 0.6 and *p* < 0.01 to improve accuracy. A random forest model was used to compare the results, and duplicate items were retained. The random forest algorithm adopts the “Boruta” algorithm to calculate in RStudio. To improve the accuracy, we set the importance threshold of the variables to >ShadowMax. Mantel test uses dplyr and linkET program packages and sets the threshold as *r* > 0.6 and *p* < 0.01. After excluding nonsignificant factors in different datasets with the above algorithm, the composite structural equation model was used to fit the relationship between variables in the four data sets, which was performed using the *nlme*, *lme4*, *piecewiseSEM*, and *QuantPsyc* packages. The above method can be referred to Tan et al. and Liu et al. [92, 93]. To determine the relative contribution rates of metabolites, microorganisms, and soil factors to the percentage of Cd aggregation hotspots, variance partial analysis was used to quantify the explanatory amount of single data factors and their interactions on the percentage of Cd aggregation hotspots.

## AUTHOR CONTRIBUTIONS

**Hong‐Rui Wang**: Conceptualisation; methodology; software; formal analysis; investigation; writing—original draft. **Xin‐Ran Du**: Software; formal analysis; investigation. **Zhuo‐Yun Zhang**: Methodology; software; formal analysis. **Fu‐Juan Feng**: Writing—review and editing; funding acquisition. **Jia‐Ming Zhang**: Software; formal analysis.

## CONFLICT OF INTEREST STATEMENT

The authors declare no conflict of interest.

## Supporting information

Supporting information.

Supporting information.

## Data Availability

All raw sequence data and code in this study are available from the corresponding author on request. Nucleic acid sequence data have been submitted to the National Centre for Biotechnology Information (NCBI) Sequence Read Archive under Bioproject PRJNA975279 https://www.ncbi.nlm.nih.gov/sra/?term=PRJNA975279/. Supplementary materials (figures, tables, scripts, graphical abstract, slides, videos, Chinese translated version and update materials) may be found in the online DOI or iMeta Science http://www.imeta.science/.
