# Supplementary material for: Rhizosphere interface microbiome reassembly by arbuscular mycorrhizal fungi weakens cadmium migration dynamics
Source: Imeta. 2023 Aug 31;2(4):e133. doi: 10.1002/imt2.133 (PMC10989832; doi:10.1002/imt2.133)
Supplement: Supplementary file 1 — Supporting information. [file IMT2-2-e133-s002.docx]

**SUPPORTING INFORMATION TO:**

**Rhizosphere interface microbiome reassembly by arbuscular mycorrhizal fungi weakens cadmium migration dynamics**

**Running title: Arbuscular mycorrhizal fungi weakens cadmium migration**

Hong-Rui Wang^1^, Xin-Ran-Du^1^, Zhuo-Yun Zhang^1^, Fu-Juan Feng^1*^, Jia-Ming Zhang^1^

^1^ Northeast Forestry University, Harbin, China

*Corresponding author

Fu-Juan Feng

E-mail：ffj9018@sina.com

**Methods and Materials**

**Concentration selection basis**

There are three reasons for the use of this concentration. First, the tolerance limit of cadmium in alfalfa was evaluated in the previous experiment, and its semi-lethal dose (EC_50_) was about 2.033 ± 0.53 mg/kg [1]. Second, this concentration is in line with the upper limit of the global average level of cadmium pollution in agricultural soil, namely 0.01-2mg/kg. Third, it just meets the severe pollution standard in the "Soil Environmental Quality Agricultural Land Soil Pollution Risk Control Standard". The pollution concentration will not damage most crops, so it is easy to cause heavy metals to accumulate in the food chain and harm human health. The purpose of this study is to prevent Cd from transferring to the above-ground parts of plants. Therefore, 2mg/kg Cd was chosen to be applied in this experiment.

[1] Wang, Hong-Rui, Xin-Yu Zhao, Jia-Ming Zhang, ChangLu, Fu-Juan Feng. 2022. “Arbuscular mycorrhizal fungus regulates cadmium accumulation, migration, transport, and tolerance in Medicago sativa.” *Journal of Hazardous Mateials* 435: 129077. <https://doi.org/10.1016/j.jhazmat.2022.129077>

**Meta-analysis method**

An extensive literature search was conducted using Google Scholar, Web of Science, PubMed, and CNKI databases from 1988 to January 2023. The search strategy combined soil microorganisms, OTU, and arbuscular mycorrhizae. A total of 2873 published articles (original research papers, except for meta-analyses) were retrieved and screened according to the following criteria: one control group and one experimental group, plants with naturally growing and inoculated arbuscular mycorrhizal fungi (including natural communities), and plant species statistics. The soil environmental parameters included nutrient content (N, P, and K), total carbon, SOM, stoichiometry (C: N, N: P), pH, and moisture. Microbial-related indicators: microbial community diversity (based on 16s amplicon sequencing). Finally, 34 parallel trial articles from 31 research papers were obtained, and the following criteria were used to select the relevant studies (Table S5):(1) Statistical information on arbuscular mycorrhizal species and the infection rate was included; (2) At least one microbial community index was reported, including the diversity index (OTU, Chao, ACE, or Shannon index); (3) Soil pH, soil carbon, soil organic matter, soil total nitrogen, soil total phosphorus, soil total potassium, soil water content, annual mean temperature (MAT), annual mean precipitation (MAP) and other data were reported. (4) The study included spatial information (latitude and longitude), and (5) ensured that the inoculated and uninoculated plants have the same growing environment. The selected dataset covered highly available biodata for microbiome and ecosystem types. The soil bacterial species that showed significant differences between the AMF and non-AMF treatment groups in the above 34 groups were counted, and the coincidence rates of functional bacteria (PGPR and HM-Remover) added to the rhizosphere soil in the AMF and non-AMF treatment groups were calculated. The natural logarithm transformation of environmental variables and microbial diversity response ratio (lnRR) of each group of parallel experiments is as described above (Zhou et al., 2020) and calculated according to the formula:

lnRR=ln(Xt− Xc)

where Xt and Xc are the microbial information and soil parameters without AMF inoculation (control group) and with AMF inoculation (treatment group), respectively. Microbial diversity was analysed using richness and Shannon indices. In the published literature, microbial richness is usually measured using OUT, Chao1, and ACE. The fixed effects model was used to detect the differences in RR for OUT, Chao, and ACE among each group. To this end, we extracted the mean standard deviation (SDs) and sample size (n) from published studies. If the standard error (SE) was reported instead of the standard deviation, the standard deviation was calculated using the following formula:

SD = sE√n

The total lnRR (including lnRR of OTUs, host plant species, pH, and microbial alpha diversity) and the corresponding target variables were calculated for the two treatments (inoculated and uninoculated AMF). The mixed effect model was used to compare the effect size of different factors on ‘functional strains’. In these analyses, groups with small sample sizes were excluded. If the 95% confidence interval of lnRR overlapped with 0, the response of the functional strains recruited by AMF to this variable was not significant. We used a non-parametric bootstrapping method, with each model iterated 500 times and sampling replacement in each interaction (each set/combination comprised two fixed effects, the interspecies interaction pattern, and the ecosystem type). The marginal mean values of each group were extracted by running the bootstrap model, and the results of fitting the mean bootstrap model without intercepts were obtained [27]. A random forest model was used to calculate the effects of soil and climate variables on the species of functional bacteria recruited by the AMF. The ‘Boruta’ algorithm in R language was used for calculation. To improve the accuracy, the importance threshold of the variables was set to < ShadowMax. SPSS (version 23.0) was used to calculate the InRR, Microsoft Excel was used for data collection and classification, and Origin 2019b was used for visual plotting.

**Rhizosphere secretion extraction experiments**

To verify that the key compounds identified above can be released into the extraneous environment in large quantities, rhizosphere exudate extraction experiments were conducted. The alfalfa treated with AM and CK were transplanted into a hydroponic solution. Move to incubators containing 0.5x Hoagland nutrient solution (20cm x25cm x15cm) for further cultivation. Paste tinfoil around the incubators to keep the roots away from light and algae blooms. Cover the top of the boxes with foam plates and seal them with foam. During the culture period, the room temperature of the photo culture room was 22-25 °C, the relative humidity was 60%-80%, and the light intensity was 5000 lx for 10h from 8:00 to 18:00 every day. At the same time, small air pumps provide the same level of oxygen to each incubator. When cultured to 21 days in hydroponics, XAD-4 adsorption resin (Suolaibao, Beijing) was soaked in 75% ethanol and 100% ethanol and the residual ethanol was rinsed with ultra-pure water for several times, and the root exudates were placed in the culture medium for collection and enrichment. After the root secretion was collected by resin for 72h, the eluent was eluted with a mixture of methanol and ethyl acetate. The eluent was dried by anhydrous Na2SO4, concentrated by rotary evaporation, and dissolved in 2mL methanol. After the filtration membrane with a 0.22μm needle, the eluent was placed in a brown sample bottle. The secretion was also determined using LC-MS. As shown in the following figure.


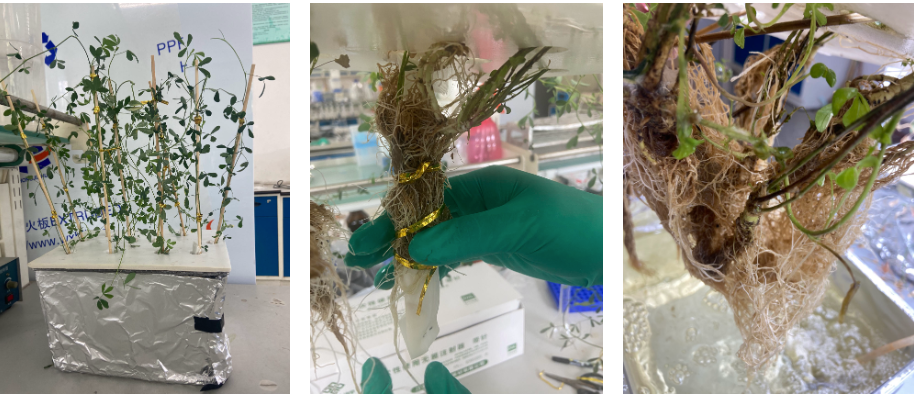


**Supplementary Figures**


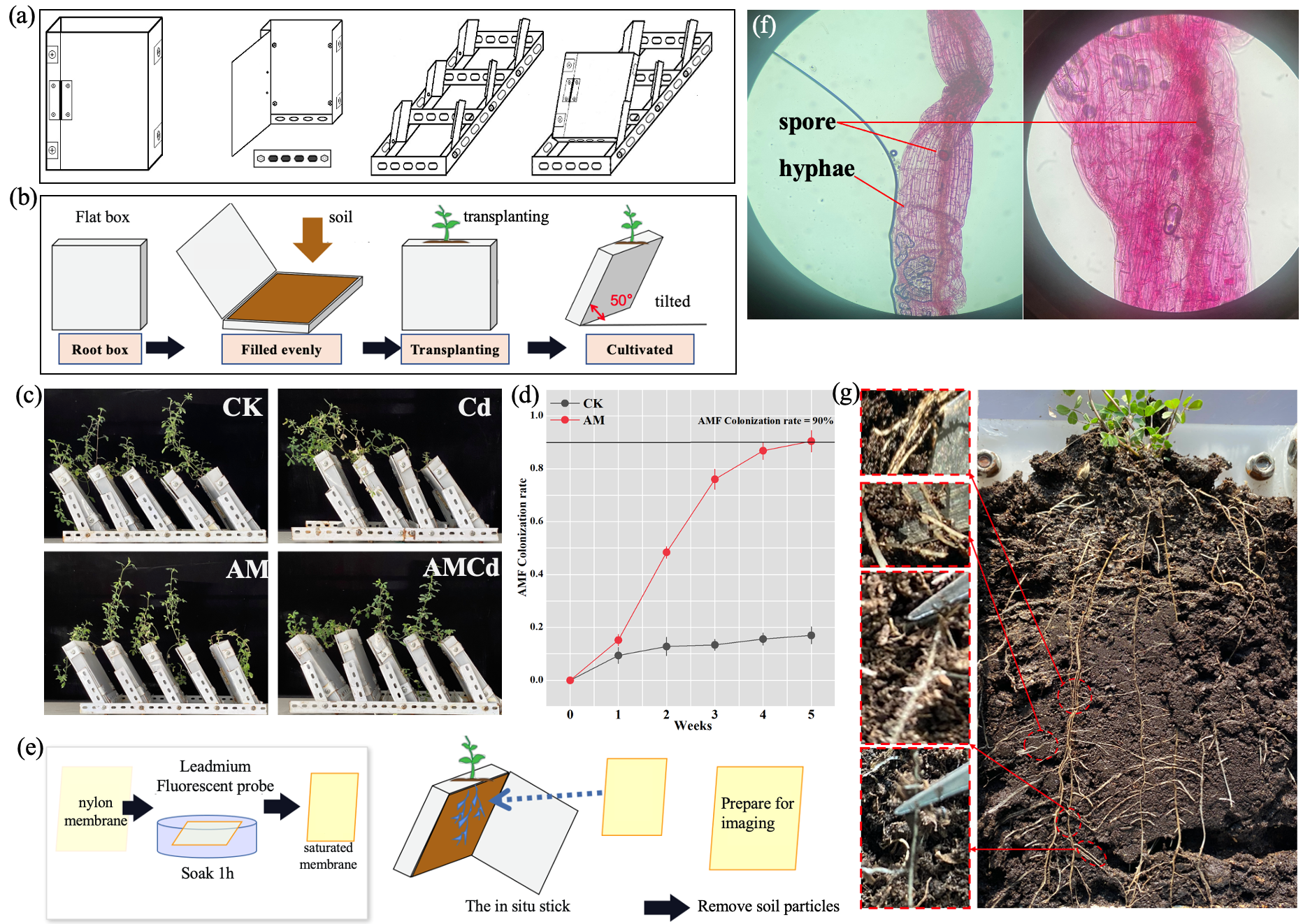


Figure S1 Procedure of root box experiment and Cd fluorescence blotting. (A) Design of root box and root box stand. (B) Alfalfa seedlings transplanted to root box and culture process. (C) Growth of alfalfa in root box under four treatments. (D) AMF infection rate of alfalfa root. (E) Rhizosphere Cd fluorescence blotting experiment process. (F) Structure of AMF spores and hyphae in alfalfa roots under an anatomical microscope. (G) Rhizosphere soil sampling display.


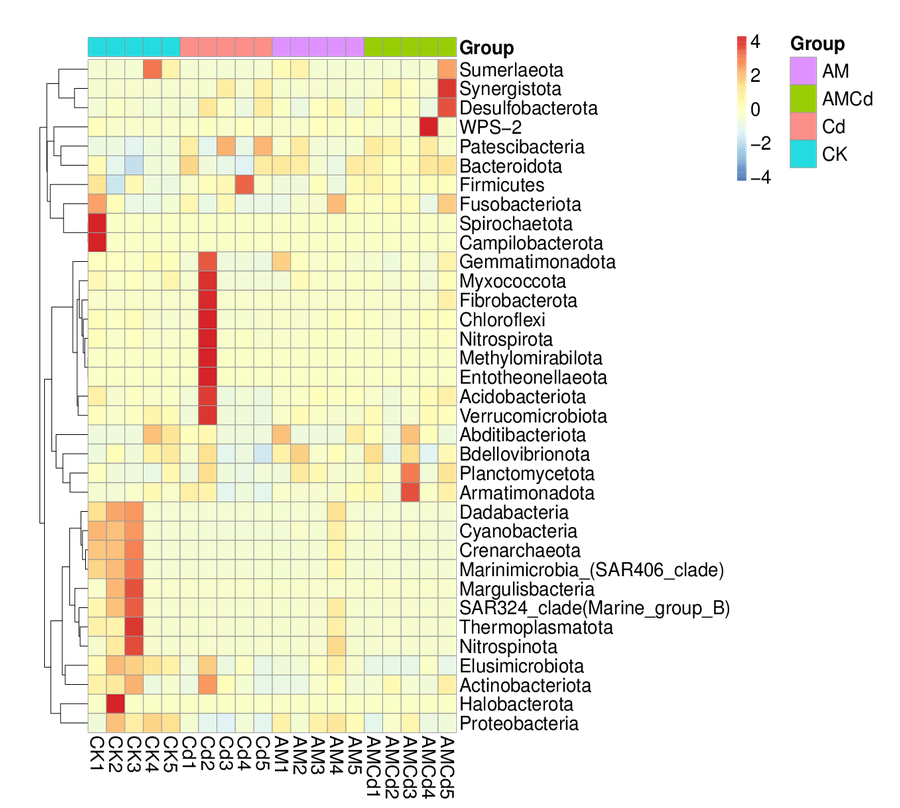


Figure S2 Based on the species annotations and abundance information of all samples at the phylum level, the top 35 genera with the highest abundance were selected according to their abundance information in each sample.


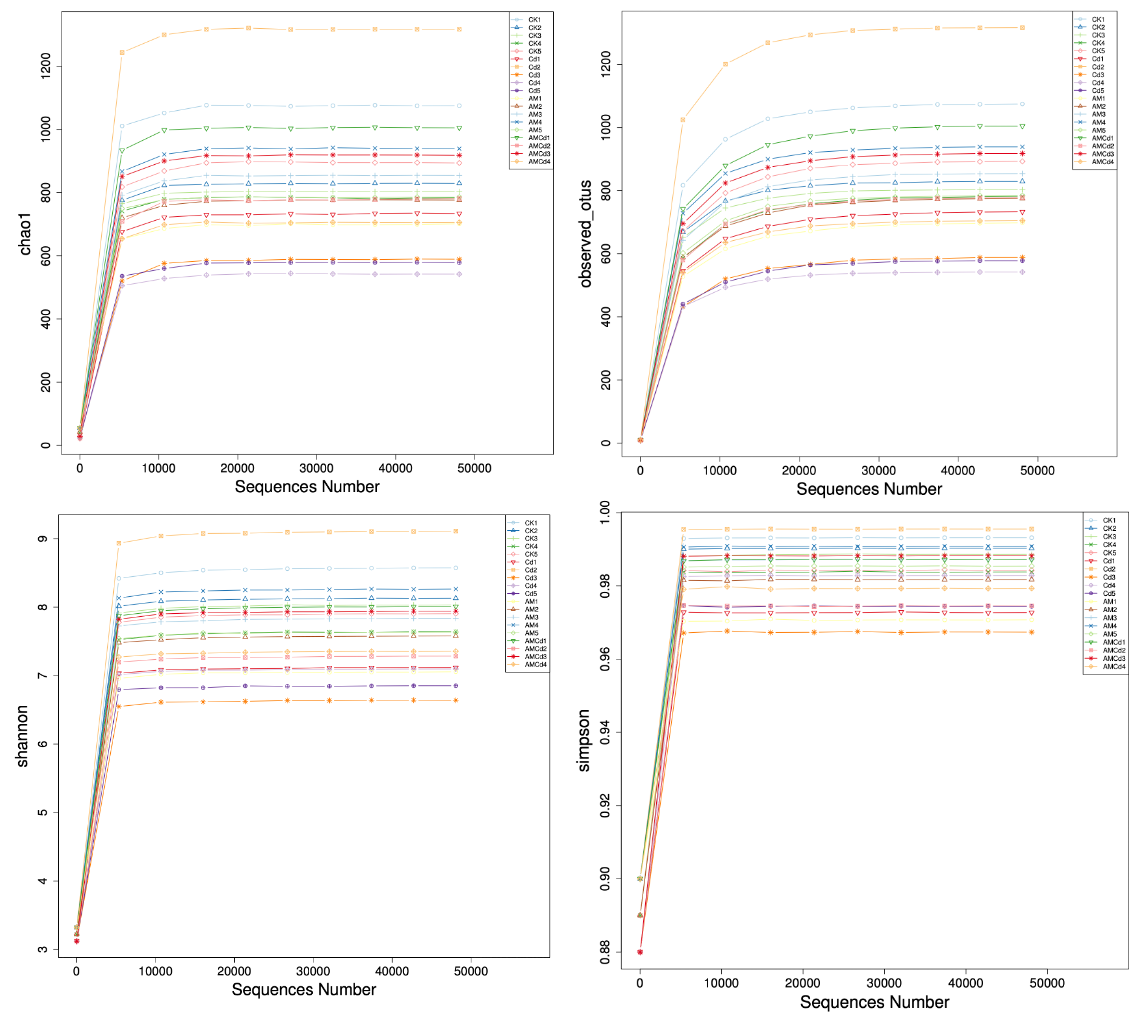


Figure S3 Rarefaction Curve is a common curve to describe sample diversity within a group. A certain amount of sequencing data is randomly extracted from samples, and their alpha diversity index values are counted. A curve is constructed with the extracted sequencing data and corresponding index values (cutoff = 48052). The horizontal axis represents the amount of sequencing data and the vertical axis represents the corresponding alpha diversity index. When the curve tends to be flat, it indicates that the amount of sequencing data is progressive and reasonable, and more data will not have a significant impact on alpha diversity index.


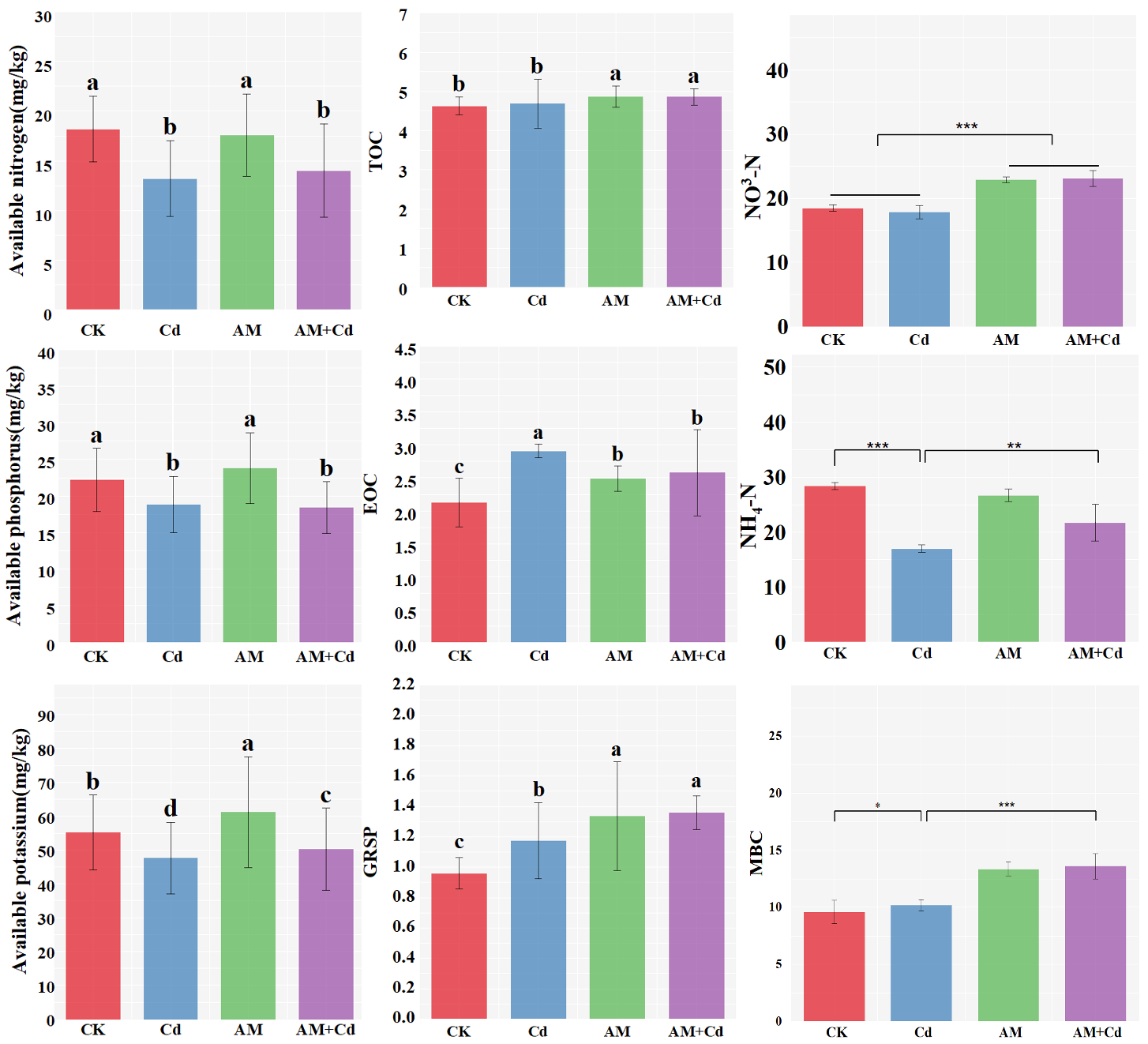


Figure S4 Changes of soil properties including available nutrients (N, P, K), N form and C component with different treatments for CK, Cd, AM, AM+Cd. Five biological replicates were performed for each index, and the error bars are standard errors. Different letters indicate significant differences (*p* < 0.05) among each treatment and refer to each subset of data within each sampling date.


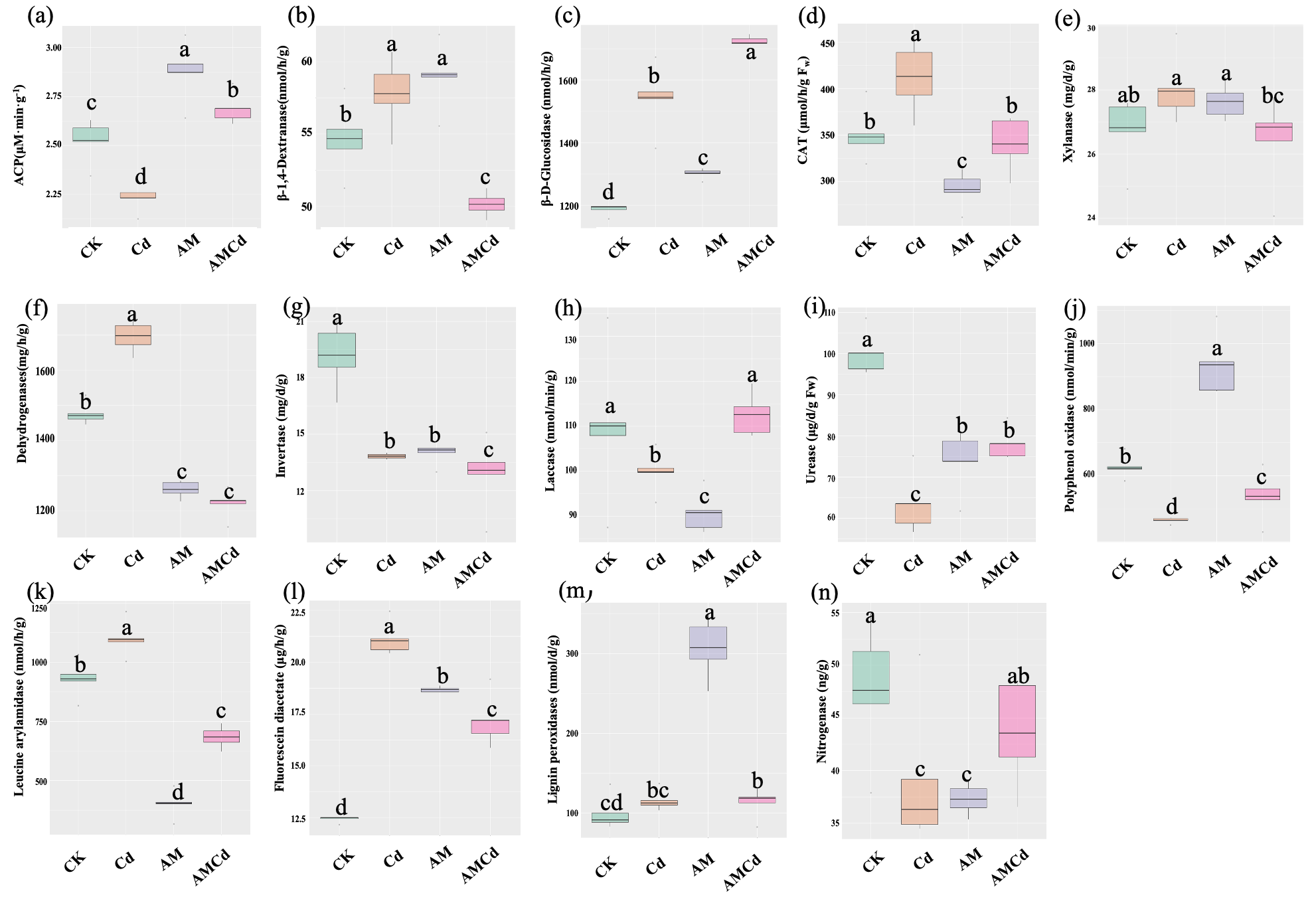


Figure S5 Changes of soil extracellular enzyme activity with different treatments for CK, Cd, AM, AM+Cd. Five biological replicates were performed for each index, and the error bars are standard errors. Different letters indicate significant differences (*p* < 0.05) among each treatment and refer to each subset of data within each sampling date.


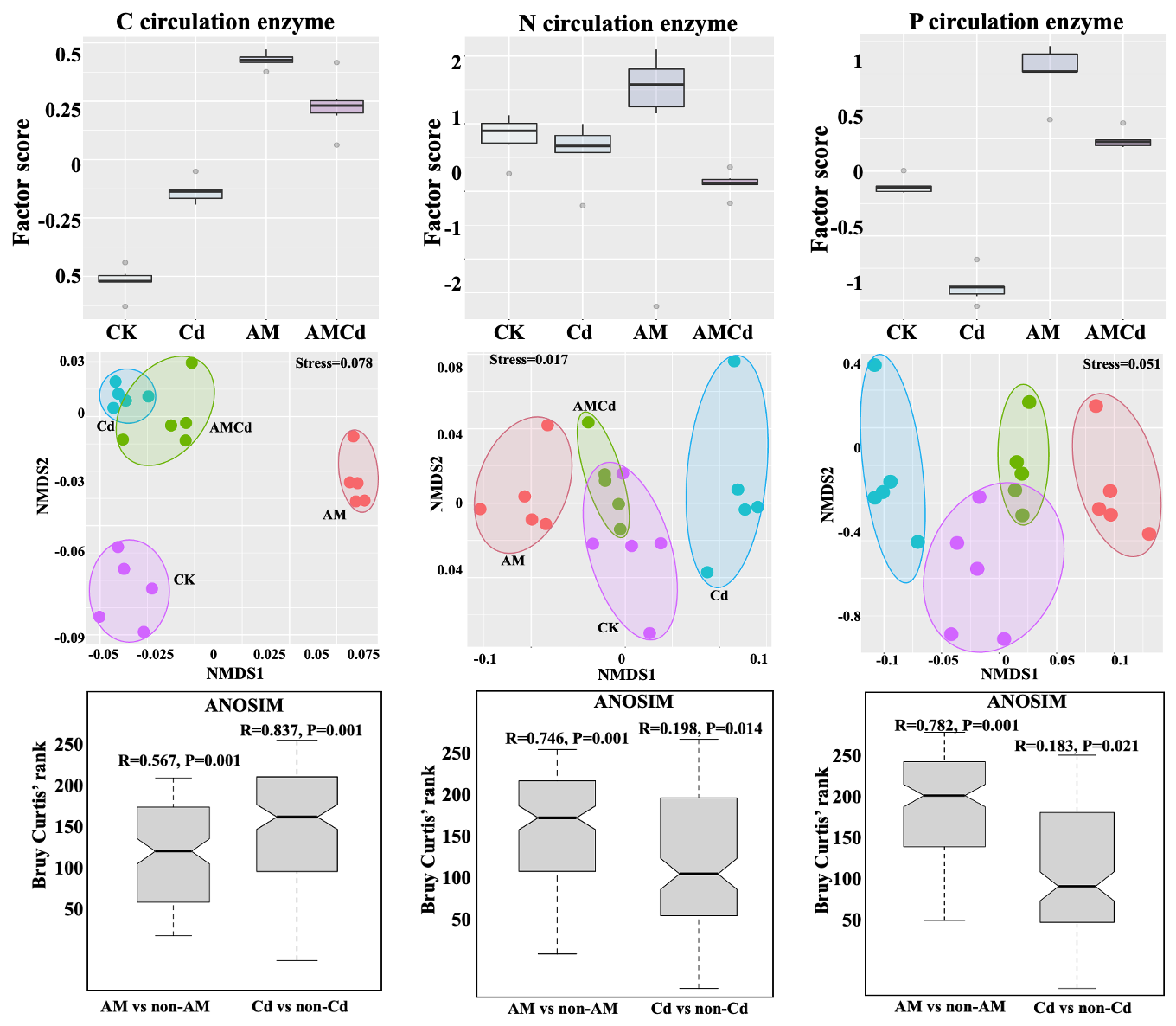


Figure S6 Score the function of C, N and P cycles function with different treatments for CK, Cd, AM, AM+Cd.


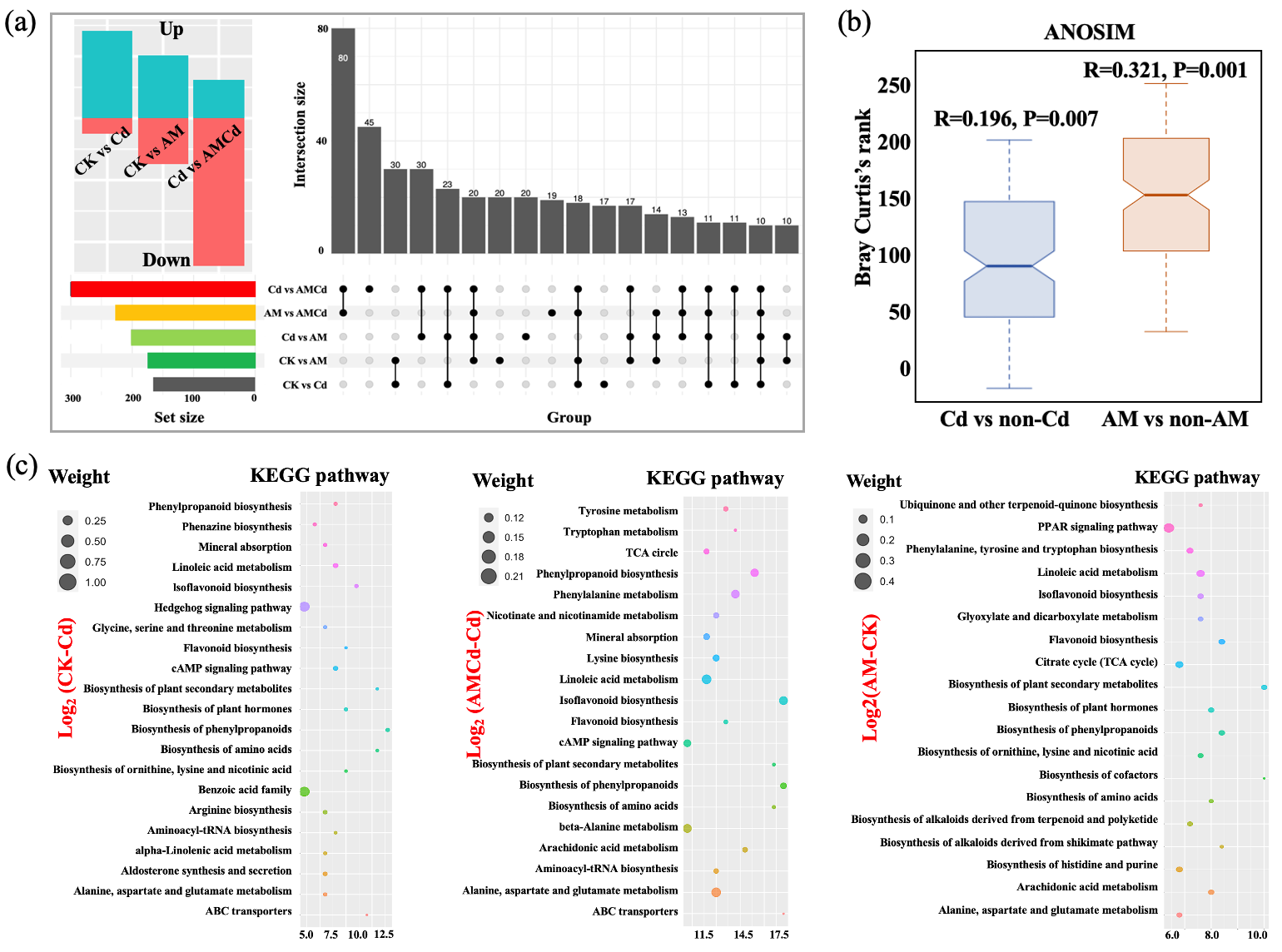


Figure S7 Changes of alfalfa root metabolism under four treatments for CK, Cd, AM, and AM+Cd. (A) Cluster analysis is used to determine metabolic patterns of metabolites under different experimental conditions. Metabolites with similar metabolic patterns have similar functions or are involved in the same metabolic process or cellular pathway. (B) An Upset Venn diagram of the number of different metabolites common and unique in different alignment groups. The numbers in the bar chart show the number of metabolites. (C) Differential metabolite pathway analysis based on the KEGG database in different alignment groups. The horizontal axis is the difference in substance expression of the experimental groups compared in pairs and Weight is the influence value of the metabolic pathway.


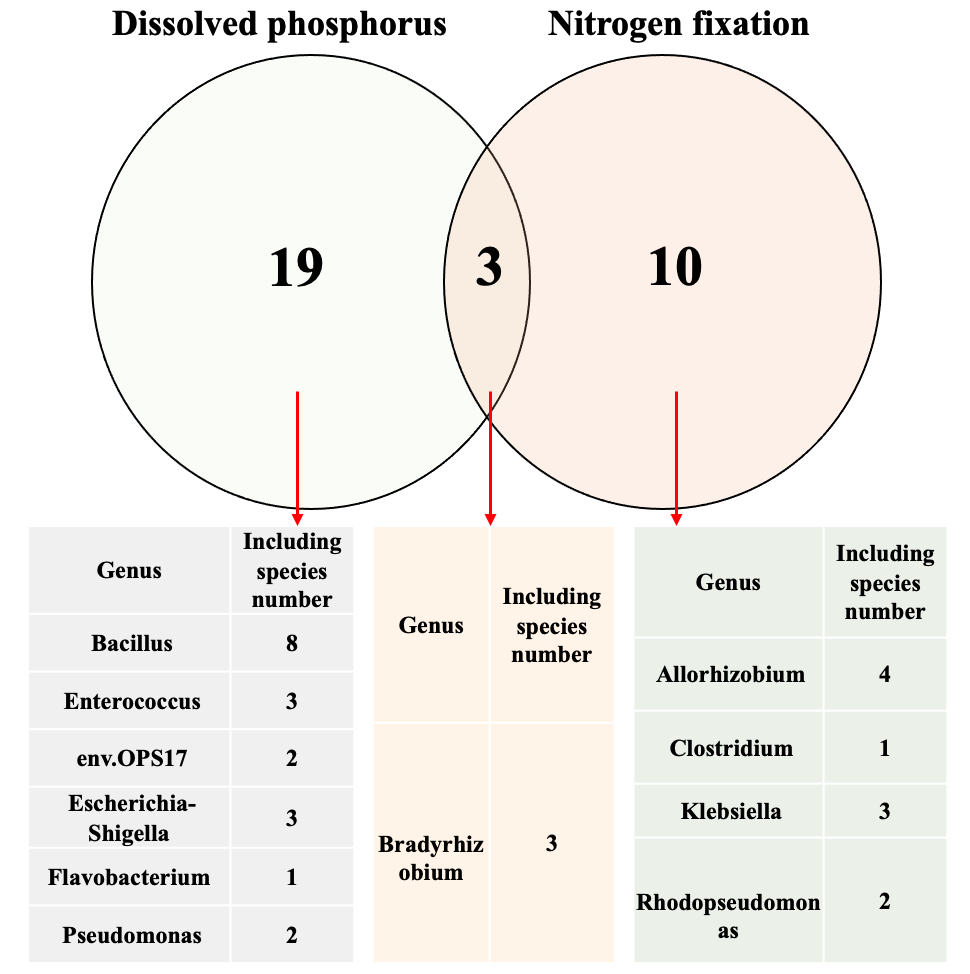


Figure S8 PGPR was screened from HM-Remover and its functions were divided.


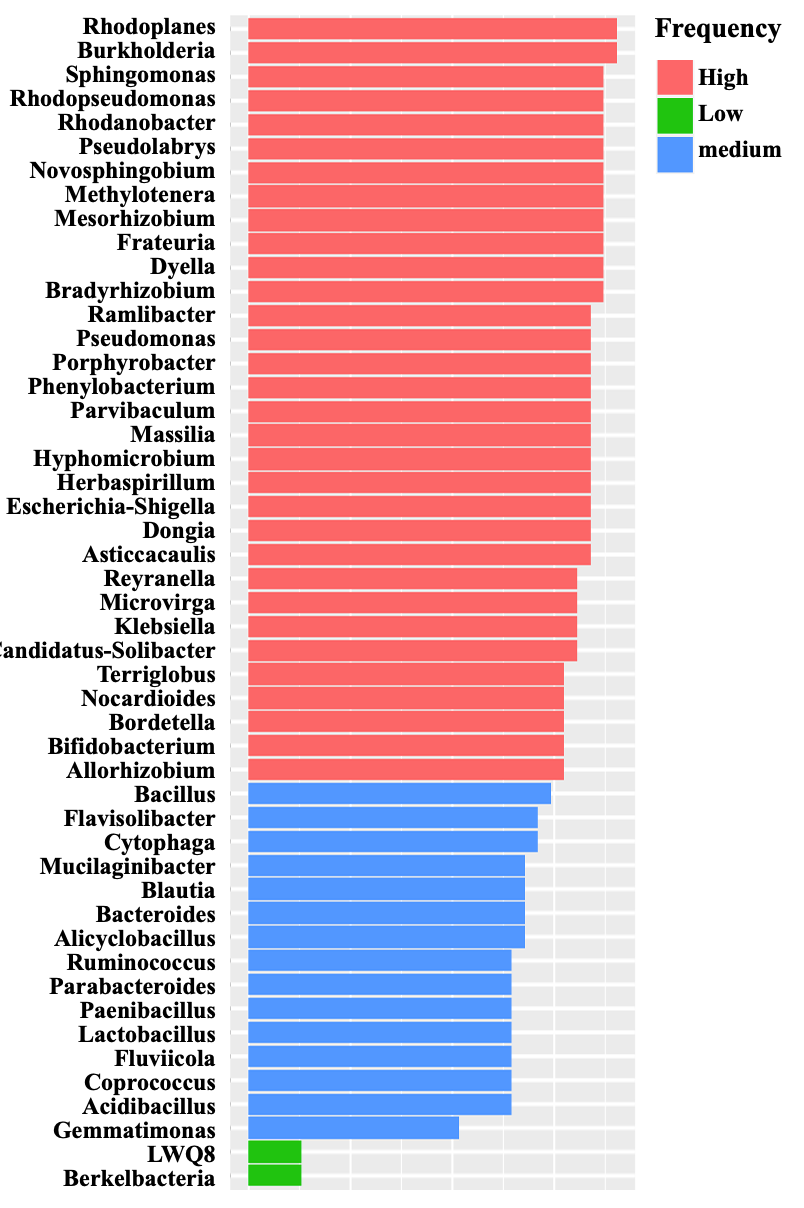


Figure S9 Meta-analysis was conducted to compare the statistics of occurrence frequency of HM-Remover types recruited by AMF in the experiment. Those with more than 20 microbial occurrences are defined as high-frequency species, those with more than 10 but less than 20 microbial occurrences are defined as medium-frequency species, and those with fewer than 10 microbial occurrences are defined as low-frequency species. The classification is based on the quartile of total occurrences.


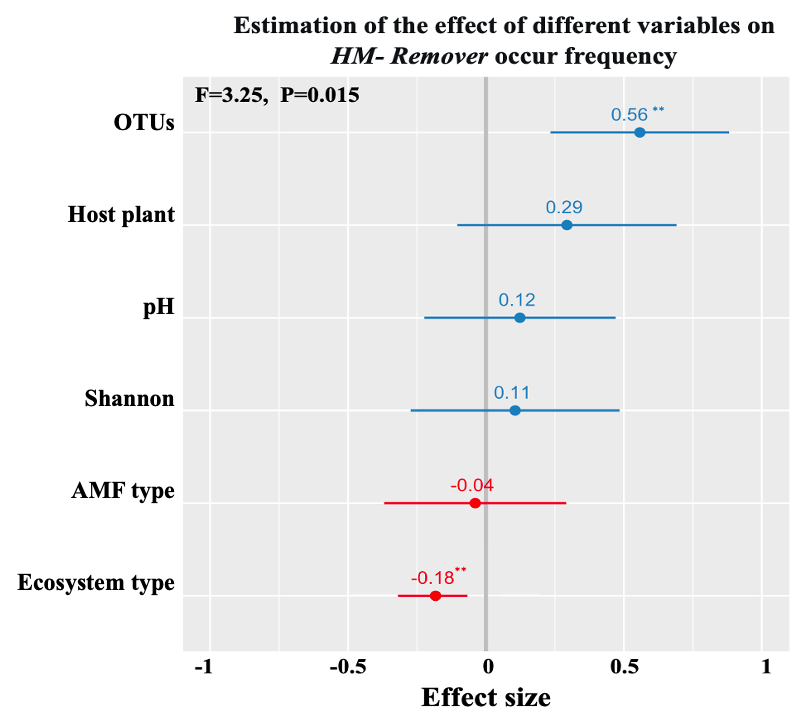


Figure S10 a Random effects model between HM-Remover occur frequency and potentially correlated variable. The significance of soil environment factors and biome types is tested by the F-test. Weighted means and their 95% confidence intervals of RRs are given.


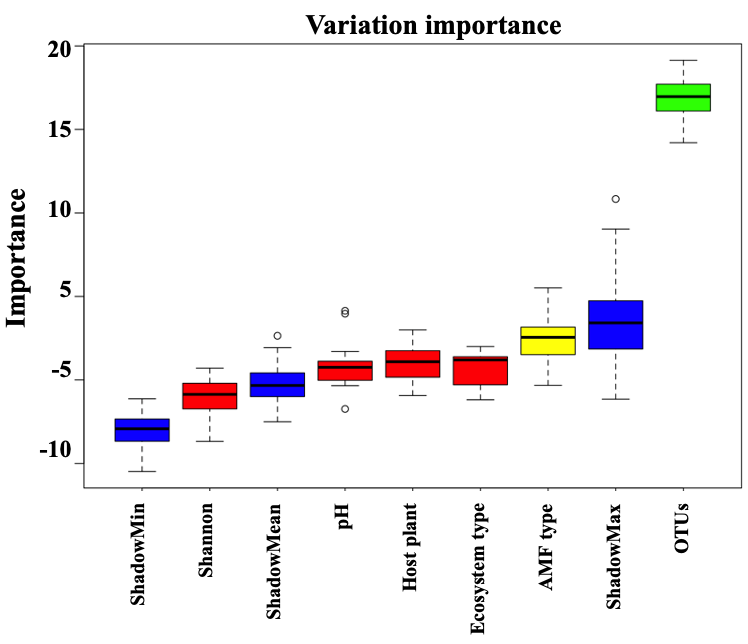


Figure S11 a Random forest model between HM-Remover occur frequency and potentially correlated variable.


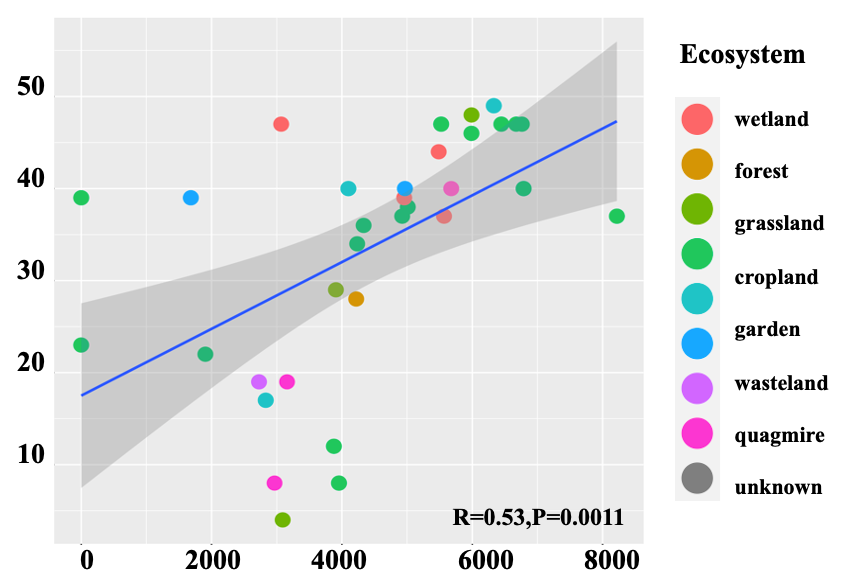


Figure S12 Linear relationships between HM-Remover occur frequency and OTUs (Regardless of ecosystem differences). The x-coordinate is the number of OTUs at this sampling point, and the y-coordinate is the frequency of HM-Remover in this test.


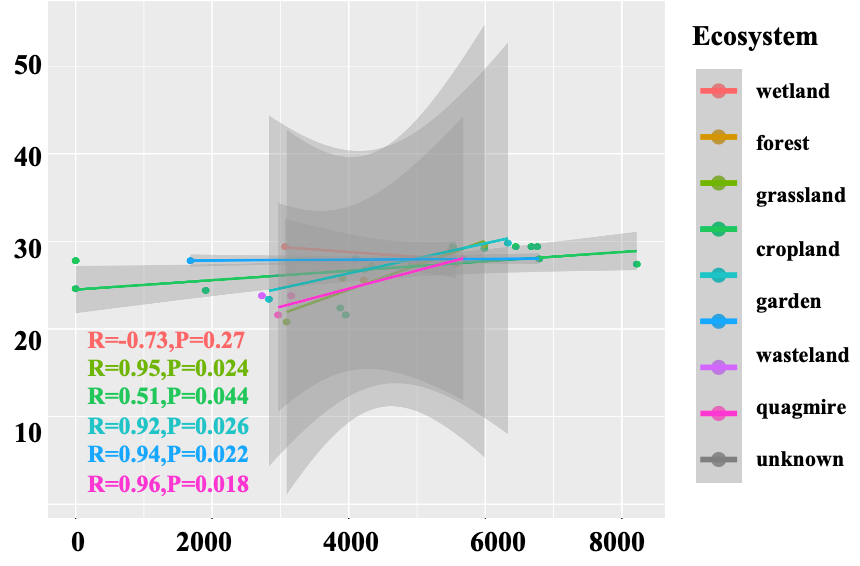


Figure S13 Linear relationships between HM-Remover occur frequency and OTUs under different ecosystem type. The x-coordinate is the number of OTUs at this sampling point, and the y-coordinate is the frequency of HM-Remover in this test.


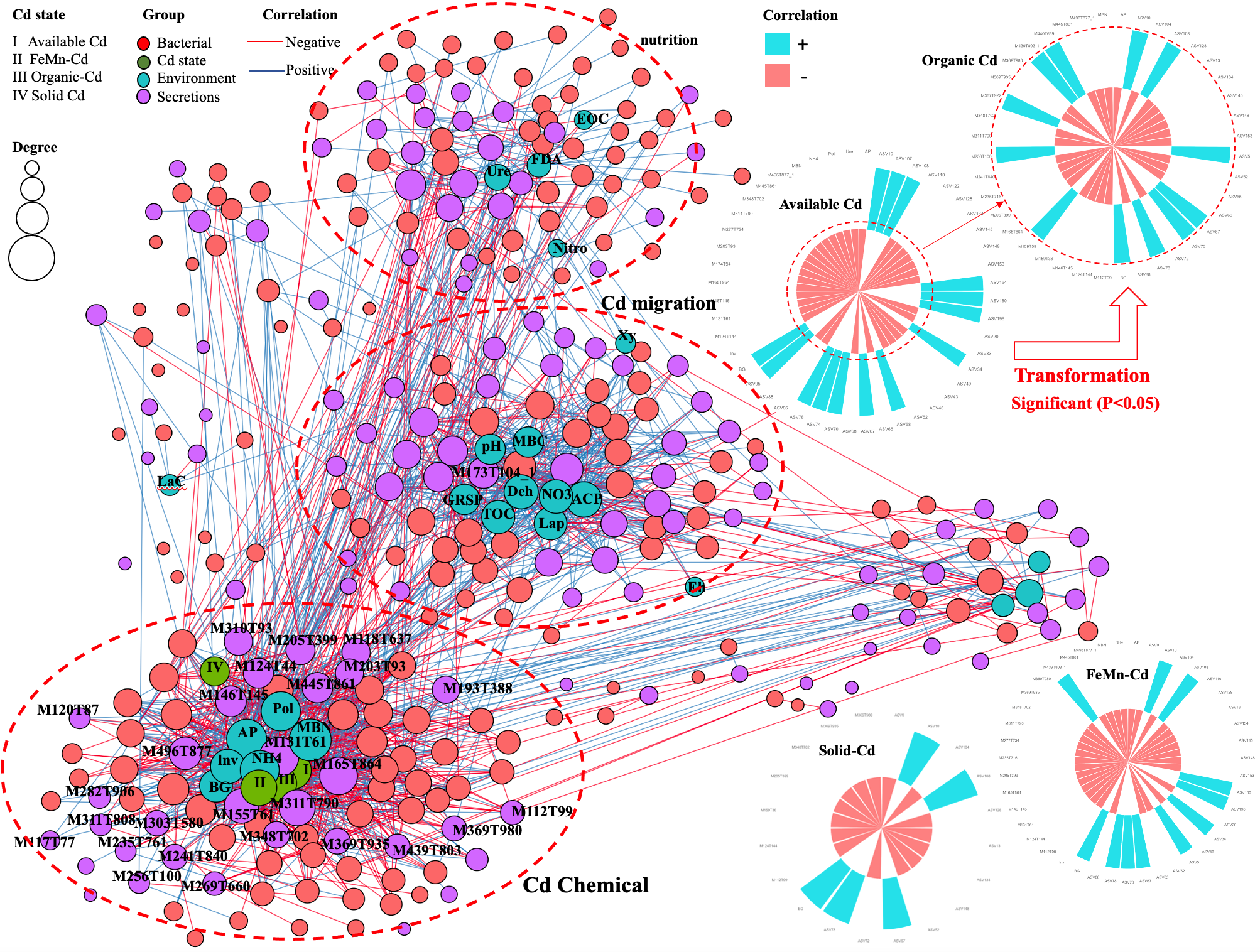


Figure S14 Modular analysis using ggClusterNet


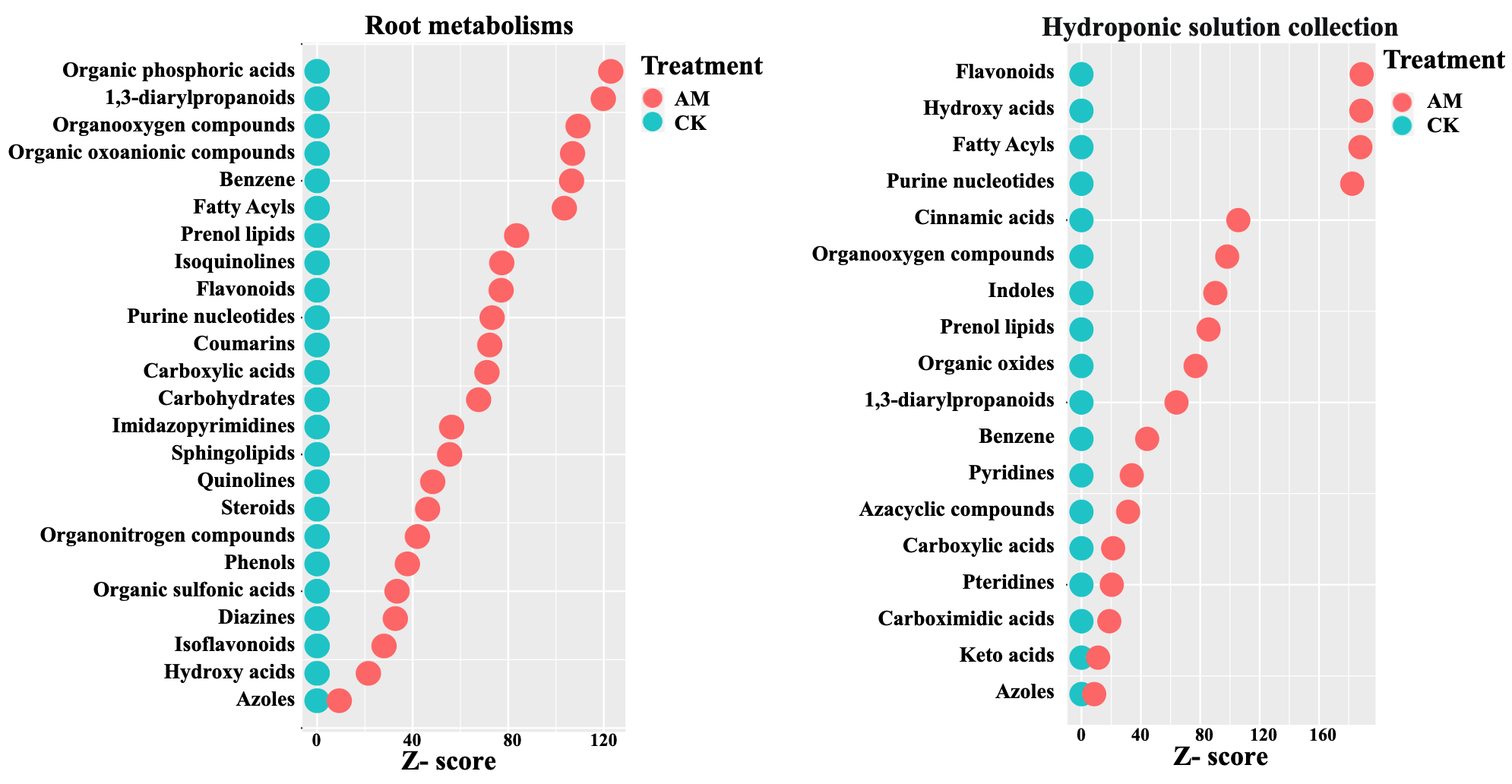


Figure S15 Comparison of endogenous metabolites and rhizosphere secretions between CK and AM
